# Recent applications of porphyrins as photocatalysts in organic synthesis: batch and continuous flow approaches

**DOI:** 10.3762/bjoc.16.83

**Published:** 2020-05-06

**Authors:** Rodrigo Costa e Silva, Luely Oliveira da Silva, Aloisio de Andrade Bartolomeu, Timothy John Brocksom, Kleber Thiago de Oliveira

**Affiliations:** 1 Departamento de Química, Universidade Federal de São Carlos, São Carlos, SP, 13565-905, Brazil; 2 Departamento de Ciências Naturais, Universidade do Estado do Pará, Marabá, PA, 68502-100, Brazil

**Keywords:** energy transfer, photocatalysis, photooxygenation, photoredox, porphyrins

## Abstract

In this review we present relevant and recent applications of porphyrin derivatives as photocatalysts in organic synthesis, involving both single electron transfer (SET) and energy transfer (ET) mechanistic approaches. We demonstrate that these highly conjugated photosensitizers show increasing potential in photocatalysis since they combine both photo- and electrochemical properties which can substitute available metalloorganic photocatalysts. Batch and continuous-flow approaches are presented highlighting the relevance of enabling technologies for the renewal of porphyrin applications in photocatalysis. Finally, the reaction scale in which the methodologies were developed are highlighted since this is an important parameter in the authors’ opinion.

## Introduction

In the last decade, photochemistry has re-emerged as a powerful tool for the scientific community. Although photochemical processes have been discovered over almost two centuries [1], only recently the scientific commuity has improved reactor technologies for the application of these processes on a large scale. The scalability of these processes had been limited by the requirement for small-volume batch reactors equipped with mercury vapor discharge lamps [1]. In general, the use of batch reactors on a large-scale is hampered due to the attenuation effect of photon transport (Bouguer–Lambert–Beer law) [1–3]. This effect limits the penetration of photons to only a short distance into the reaction vessel, provoking increases of the reaction time, photocatalyst loading, byproducts, overheating and so on. Notably, the use of continuous-flow reactors for photochemical applications allows us to overcome these issues, and leads to a drastic reduction of reaction time, lower photocatalyst loadings, minimization of the formation of byproducts [2] and uses visible light, which is considered a clean reagent [4]. Overall, visible light combined with organic photocatalysts such as porphyrinoids, make continuous-flow photochemistry a sustainable alternative approach being applied already by the chemical and pharmaceutical industries.

**Figure 1:**
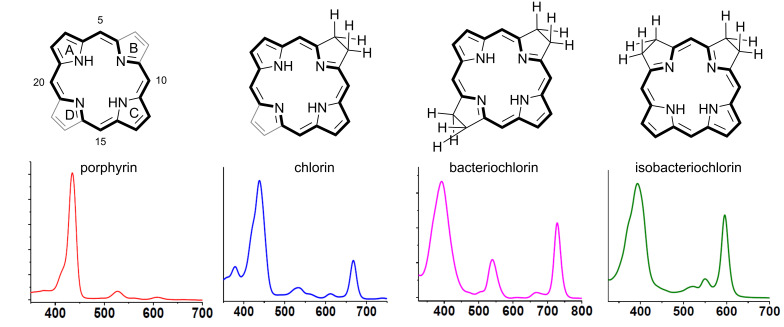
Chemical structures of the porphyrinoids and their absorption spectra: in bold are highlighted the 18 π aromatic system. Adapted from [7].

Porphyrinoid is the term given for a class of organic compounds containing four pyrrole rings connected by four methylene bridges, and include porphyrin, chlorin, bacteriochlorin, and isobacteriochlorin. The cores of the porphyrin and chlorin scaffolds contain respectively 22 π and 20 π electrons, whereas bacteriochlorins and isobacteriochlorins contain 18 π electrons (Figure 1). The 18 π electron aromatic system (Figure 1, in bold) of the porphyrinoids confers stability, planarity and special electronic characteristics to these compounds. As it is well-known, tetrapyrrolic compounds are considered to be the “pigments of life” since they play a key role in essential biological processes, such as photosynthesis (chlorophylls and bacteriochlorophylls), redox reactions for detoxification of anthropogenic chemicals (cytochrome P450) and oxygen transport (hemoglobin) [5–6].

Taking into account this big group of molecules, porphyrins are notable due to both their physicochemical and electronic properties, which can be fine-tuned by functionalization of the core structures [8]. The adequate tuning of the porphyrin properties can enable them to absorb light in almost all of the UV–vis spectral range. Porphyrins also have elevated molar absorptivity (ca 10^5^ L·mol^−1^·cm^−1^) and appropriate electronic levels for both energy transfer (ET) and single electron transfer (SET) in many photoprocesses [9–11]. Additionally, it is possible to realize tuning in terms of chemical properties by changing substituents, thus producing robust, soluble or heterogeneous, readily available and low-cost photocatalysts.

**Figure 2:**
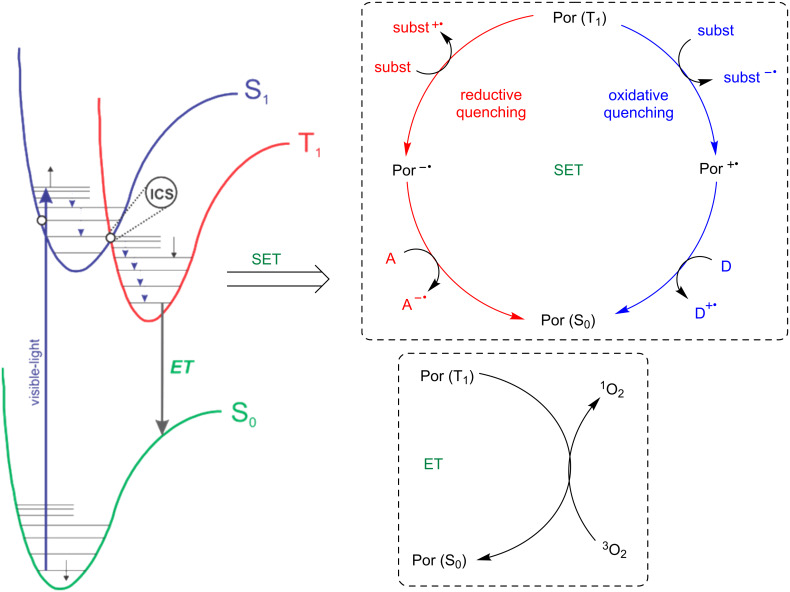
Photophysical and photochemical processes (Por = porphyrin). Adapted from [12,18].

The mechanisms of the photocatalytic activities of porphyrins are similar to other photocatalysts. Under light irradiation, one electron from the ground state (S_0_) is promoted to the excited singlet state (S_1_) which has a short lifetime (10^−9^ s). Therefore, fast intersystem-crossing of one electron gives the excited triplet state (T_1_) with a relatively longer lifetime (10^−6^ s). While the porphyrin is in the triplet excited state, two distinct processes can be observed: a) single electron transfer (SET); and b) energy transfer (Figure 2) [12–14]. The first involves the exchange of electrons between the porphyrin and the substrate by an oxidative or reductive process, and the second involves energy transfer to surrounding molecules, such as molecular oxygen, heterocycles and other relevant molecules [15–17].

The excited states of porphyrins are also both potent oxidants and reductants when compared to the ground state. This phenomenon can be measured by the comparison of the standard reduction potentials for the photocatalyst in both ground and excited states [14]. For example, the oxidation potentials for ground [*E*_1/2_(TPP^+•^/TPP)] and excited states [*E*_1/2_(TPP^+•^/TPP^*^)] of tetraphenylporphyrin (TPP), whose electrochemical data are available [10], are +1.03 V and −0.42 V, respectively (both vs saturated calomel electrode (SCE)). These data indicate that the excited state of TPP is a more efficient electron donor than its ground state. At the same time, the reduction potential value suggests that the excited state of TPP (*E*_1/2_(TPP^*^/TPP^−•^) = +0.42 V vs SCE) is a more efficient electron acceptor than its ground state (*E*_1/2_(TPP/TPP^−•^) = −1.03 V vs SCE). Thus, depending on the reaction system in which this photocatalyst is being used, reductive or oxidative processes can be accomplished.

In this review, we intend to highlight applications of porphyrins and their analogs in both photoredox and energy transfer photocatalyzed reactions. The idea of this review is also to cover representative chemical transformations and recent applications in both batch and continuous-flow conditions, and emphasizing as much as possible, the scale in which the reactions were described. It is important to clarify that other relevant reviews reporting applications of porphyrins in different perspectives can be found in the literature [19–21].

In addition, we emphasize that this review is organized into two topics. The first topic highlights the reactions that employ porphyrins as photoredox catalysts in both oxidative and reductive quenching. The use of porphyrins as a photosensitizer for singlet oxygen generation is presented in the second topic, which was subdivided into two sections: pericyclic reactions and heteroatom oxidations. The first section describes the use of singlet oxygen in pericyclic reactions with olefins and dienes, and the second deals with heteroatom oxidations carried out by singlet oxygen.

## Review

### Porphyrins as photoredox catalysts

**Figure 3:**
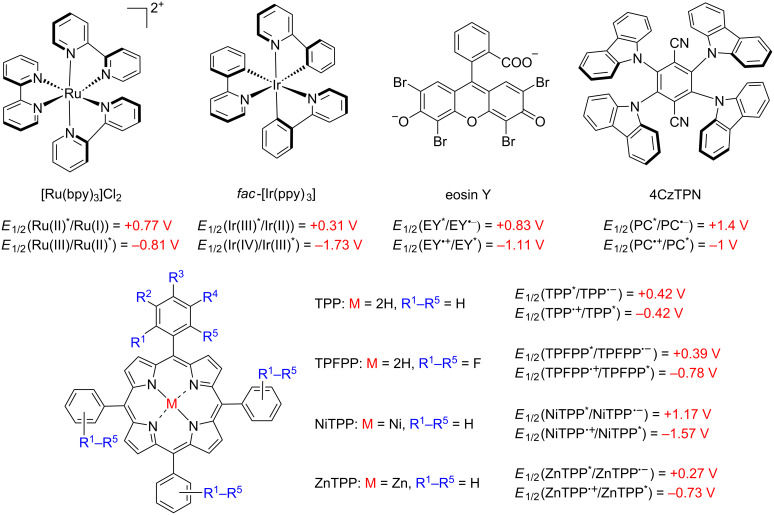
Main dual photocatalysts and their oxidative/reductive excited state potentials, including porphyrin derivatives. Adapted from [10,12,22].

Porphyrins and metalloporphyrins have been extensively studied as photosensitizers in singlet oxygen generation, but underexploited as photoredox catalysts up to now [10,22]. Remarkably the same system can be applied for both pathways, oxidative and reductive processes, beyond singlet oxygen generation [9–10,23]. Only a few photocatalysts can be applied in both photoredox processes (oxidative and reductive quenching), for example, [Ru(bpy)_3_)]^2+^, [Ir(ppy)_3_], eosin Y, and 4CzTPN [12] (Figure 3). However, some porphyrin and metalloporphyrin derivatives possess adequate potentials to be applied as photoredox catalysts in C–C and C-heteroatom bond formations [10,22]. Furthermore, supramolecular porphyrin-containing molecules, such as metal-organic (MOF) and covalent-organic frameworks (COF), have significantly expanded the use of these compounds in photoredox catalysis due to the singular electronic features of these materials and chemical robustness as catalysts.

**Scheme 1:**
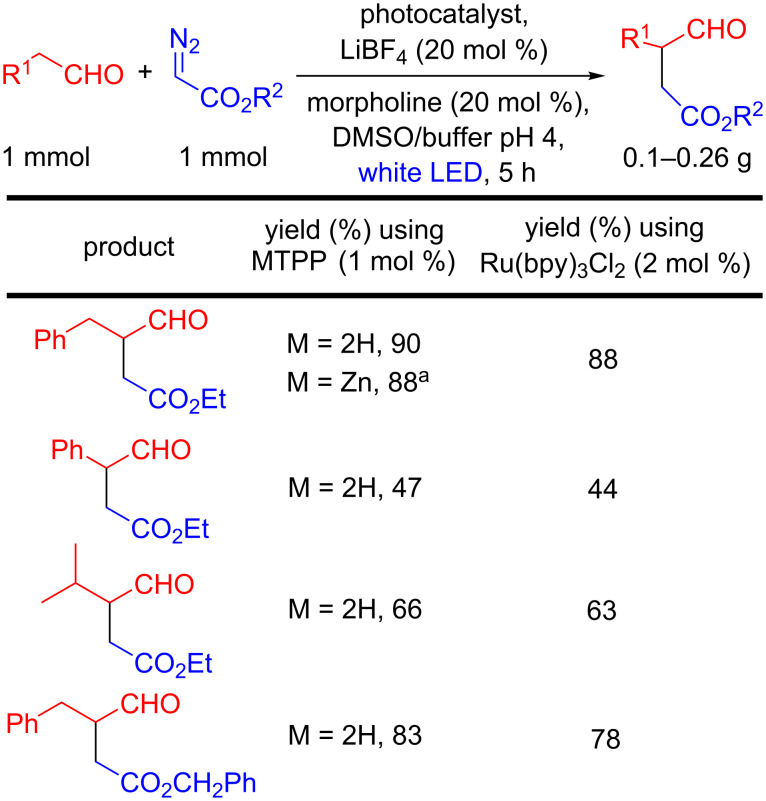
Photoredox alkylation of aldehydes with diazo acetates using porphyrins and a Ru complex. ^a^Using a loading of 0.1 mol % for ZnTPP.

The appearance of porphyrins as photoredox catalysts for C–C bond formation started in 2016 with the report from Gryko’s group on the photoredox α-alkylation of aldehydes with diazo compounds using 1 mol % of TPP or ZnTPP as photocatalyst [10] (Scheme 1), thus obtaining functionalized aldehydes in 47–90% yields. These results are similar to those previously reported by the same authors using 2 mol % of a Ru complex as photocatalyst (44–88%) [24].

**Scheme 2:**
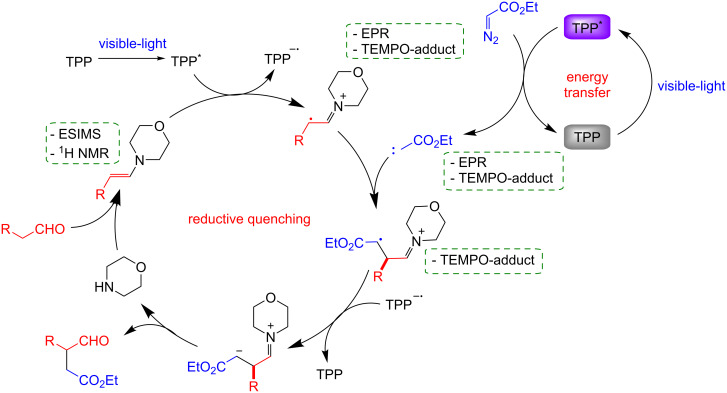
Proposed mechanism for the alkylation of aldehydes with diazo acetates in the presence of TPP.

The mechanism proposed by the authors was supported by the detection of some reaction intermediates, and suggest that TPP works in both energy transfer and photoredox catalysis (Scheme 2).

**Scheme 3:**
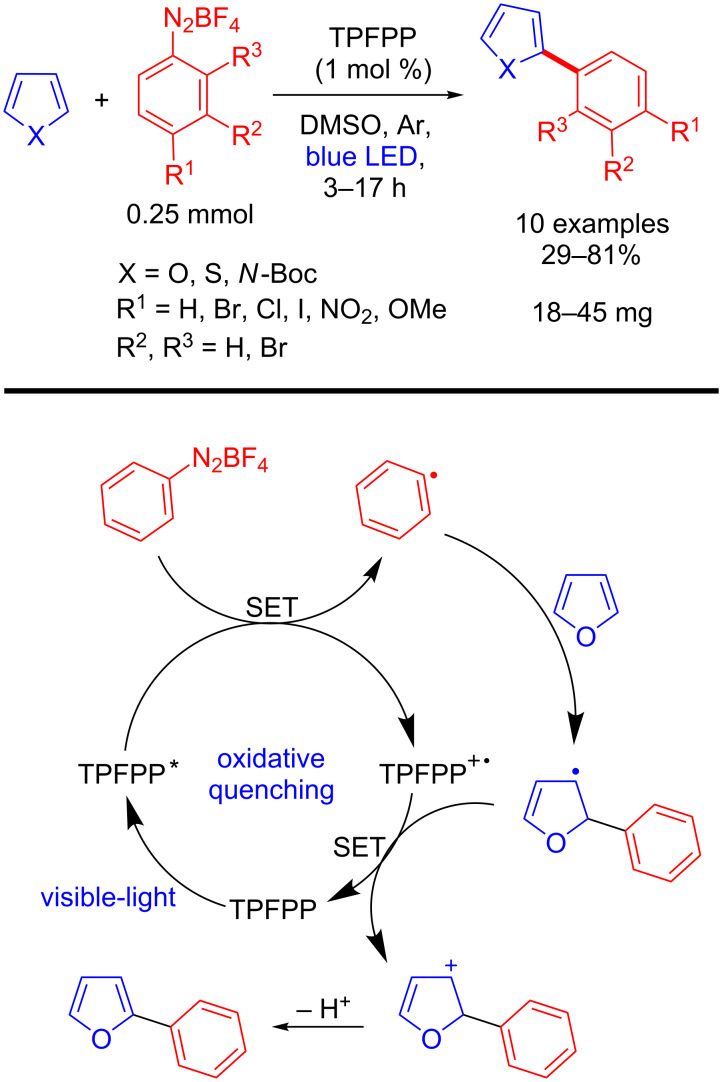
Arylation of heteroarenes with aryldiazonium salts using TPFPP as photocatalyst, and corresponding mechanism.

Subsequently, Gryko’s group reported a metal-free photoarylation of five-membered heteroarenes with aryldiazonium salts and *meso*-arylated porphyrin derivatives as photoredox catalyst [11]. Compounds such as furan, thiophene, and *N*-Boc-pyrrole derivatives were obtained by this methodology in 29–81% yields (Scheme 3). The key-step of this transformation involves the formation of an aryl radical by SET between the diazo compound and the porphyrin in its excited state (Scheme 3). The authors demonstrated that *meso*-arylated porphyrins can efficiently act by an oxidative quenching. However, issues about why an electron-poor porphyrin such as tetrakis(pentafluorophenyl)porphyrin (TPFPP) is more efficient in an oxidative quenching compared to an electron-rich (e.g., TPP) remained to be elucidated.

**Scheme 4:**
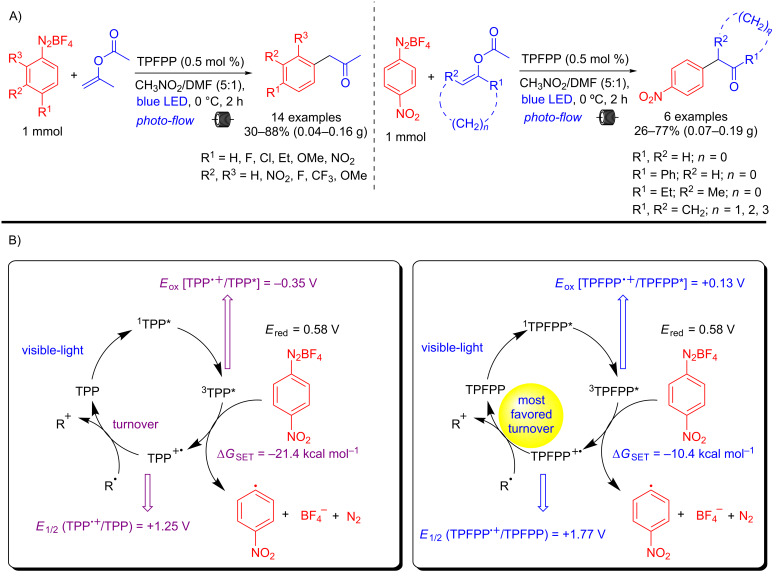
A) Scope with different aryldiazonium salts and enol acetates. B) Photocatalytic cycles and comparison between TPP and TPFPP.

In this regard, our research group has contributed in the last 10 years with new porphyrin/chlorin synthetic methodologies, and applications of these compounds in photomedicine [23,25–27]. Recently we reported a porphyrin-photocatalyzed protocol for the arylation of enol acetates and elucidated the mechanism explaining why the electron-deficient porphyrin TPFPP is more efficient than TPP in the whole process (Scheme 4A). Briefly, we have demonstrated that both porphyrins, in the excited state, are thermodynamically able to promote the first photooxidation step (Scheme 4B), however, the turnover of TPFPP^+^ to TPFPP is much more favored which justifies the acceleration of the photocatalytic cycle. In this protocol, the scope of the diazonium salts, as well as the enol acetates are reported giving versatile α-aryl ketones/aldehydes in both batch and continuous-flow conditions (20 examples in 26–88% yields) [9].

**Scheme 5:**
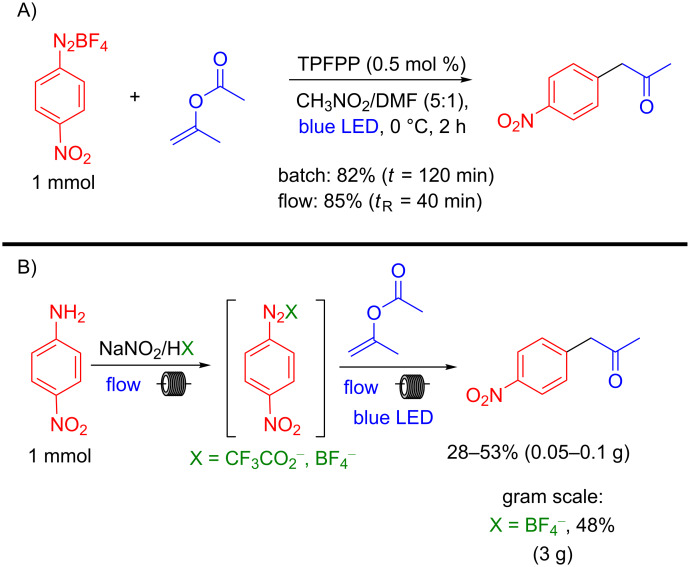
Photoarylation of isopropenyl acetate A) Comparison between batch and continuous-flow approaches and B) end-to-end protocol.

A comparison between batch and flow conditions was performed showing that similar yields are obtained (batch 82% vs flow 85%), but under continuous-flow conditions the reaction time (residence time, *t*_R_) is three times less (Scheme 5A). Our group also developed an end-to-end two-step protocol under continuous-flow conditions, in which the aryldiazonium salt was generated in situ and used directly in the photoarylation of isopropenyl acetate. The corresponding α-aryl ketone was obtained in 28–53% overall yield depending on the scale. An 8 h experiment was conducted in a continuous steady-state mode, producing the same α-aryl ketone in 48% yield on a 3 gram-scale (Scheme 5B).

**Scheme 6:**
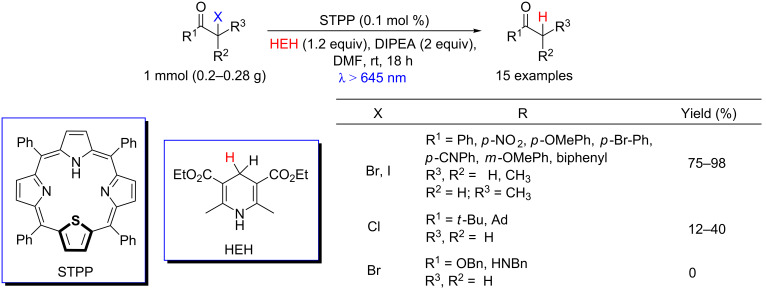
Dehalogenation induced by red light using thiaporphyrin (STPP).

Photoredox catalysis is not limited to regular N_4_-porphyrins (with four pyrrole units), but also can occur with other porphyrinoid compounds. Porphyrins containing other heteroatoms present physicochemical and electronic properties that are quite different from regular N_4_-porphyrins. These structures absorb and emit light at lower energies, as for example the thiaporphyrins that absorb beyond 650 nm [8,28]. Derksen and co-workers studied bond-cleavage reactions that can occur in biological microenvironments, using a light source with wavelengths frequently employed in photomedicine (650–850 nm) and thiophene-containing porphyrins [28]. The authors reported that *meso*-5,10,15,20-tetraphenyl-21-monothiaporphyrin (STPP), combined with Hantzsch ester (HEH) and *N*,*N*-diisopropylethylamine (DIPEA), promoted the dehalogenation of α-functionalized carbonyl-containing compounds under red light (λ > 645 nm) in a reductive quenching. DIPEA and HEH act respectively as electron and hydrogen donors. The protocol was efficient for dehalogenations with bromine- and iodine-containing acetophenone derivatives (75–98% yields). However, it was much less efficient with chloro ketones (12–40% yields) and not effective with α-bromo esters and α-bromo amides (Scheme 6).

**Scheme 7:**
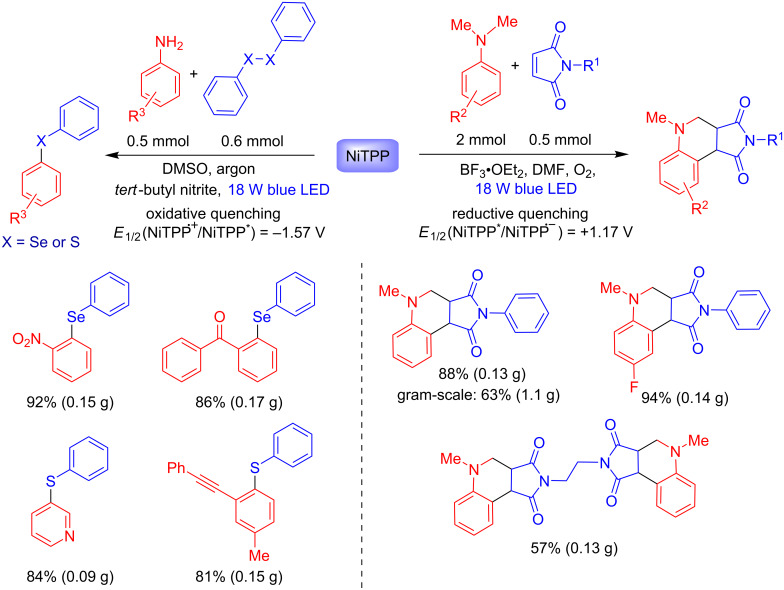
Applications of NiTPP as both photoreductant and photooxidant.

Many metalloporphyrins are applied as catalysts in industrial processes, such as in the oxidation of cyclohexane to cyclohexanone catalyzed by Co(II) tetraphenylporphyrin on a ton-scale [21]. Recently, Sarkar and co-workers reported the use of nickel(II) tetraphenylporphyrin (NiTPP) as an efficient photocatalyst in both oxidative and reductive quenching [22]. The ability of NiTPP as both photooxidant and photoreductant was observed in maleimide annulation and chalcogenylation reactions, respectively (Scheme 7). For both processes, nickel(II) was determinant for the success of these protocols, as demonstrated by the nonmetallated TPP which did not work.

**Scheme 8:**
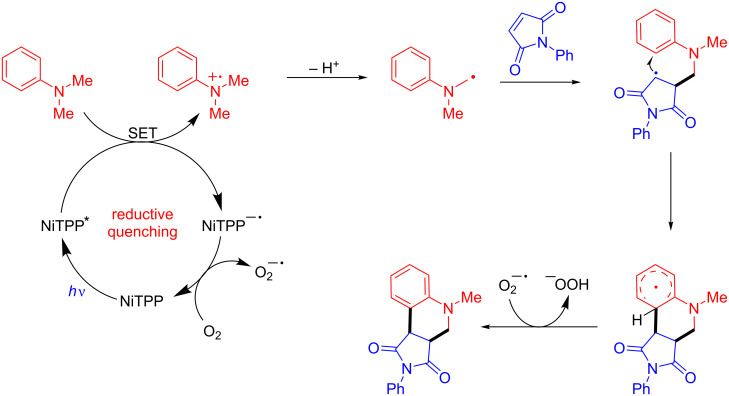
Proposed mechanism for obtaining tetrahydroquinolines by reductive quenching.

The tetrahydroquinoline products were obtained in up to 1.1 gram-scale, after 20 h under blue LED irradiation (18 W), for both *N*-alkyl/aryl maleimides (57–92% yields) and the *p*-substituted *N,N*-dimethylanilines (78–97% yields). Mixtures of regioisomers were obtained when a *m*-substituted *N,N*-dimethylaniline was used. The authors have proposed a reductive quenching pathway mechanism for this protocol (Scheme 8).

**Scheme 9:**
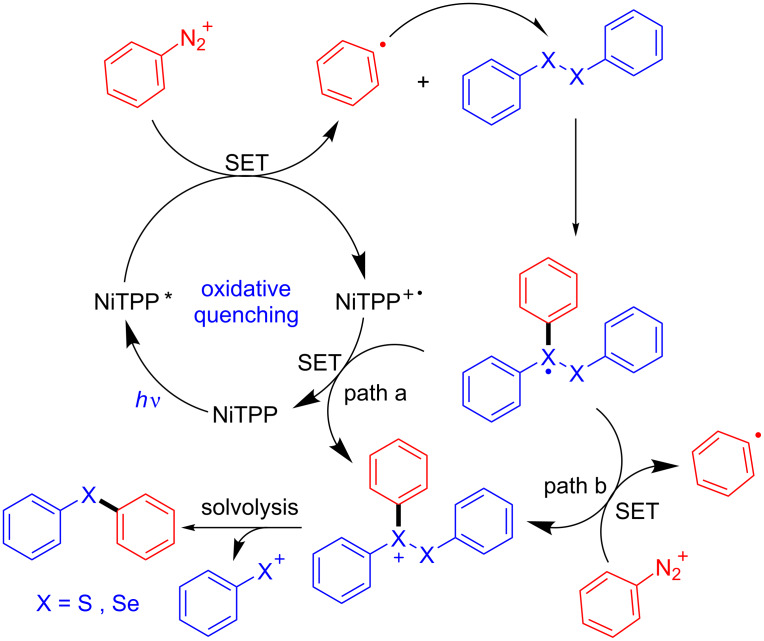
Selenylation and thiolation of anilines.

The use of NiTPP as photoreductant was also exploited in the selenylation and thiolation reactions of anilines (Scheme 7 and Scheme 9). The methodology involves the in situ formation of the aryldiazonium salt by diazotization with *tert*-butyl nitrite followed by the formation of a trivalent radical chalcogenide. Oxidation to the chalcogenide cation by SET with the cation-radical NiTPP (path a, Scheme 9) or SET with the aryldiazonium salt leads to the other aryl radical species (path b, Scheme 9). The thio- and selenoethers were obtained after solvolysis. Excellent yields (up to 94%) were reported for both selenylation and thiolation of anilines with electron-withdrawing and electron-donating groups in the *ortho*, *meta* and *para* positions. Moreover, the methodology also showed effectiveness for heteroarenes such as pyridines and benzothiazoles.

**Scheme 10:**
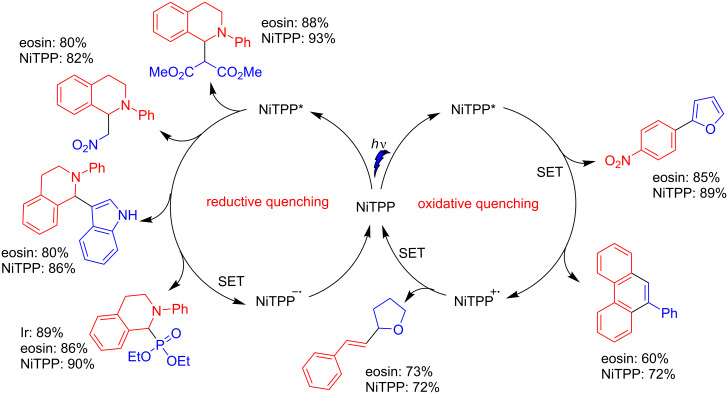
NiTPP as photoredox catalyst in oxidative and reductive quenching, in comparison with other photocatalysts.

The authors also evaluated the use of NiTPP as a photoredox catalyst for other transformations involving both oxidative and reductive quenchings. The NiTPP-catalyzed reactions between *N*-phenyltetrahydroisoquinoline and dimethyl malonate, nitromethane, indoles, and dialkyl phosphonates furnished the α-substituted *N*-phenyltetrahydroisoquinolines in yields equal or better than with the originally used photocatalysts, such as eosin and Ir-complex [29–31] (Scheme 10). For an oxidative quenching, the photoarylation of heteroarenes and alkynes with aryldiazonium salts, and the oxidative decarboxylative coupling between cinnamic acid and tetrahydrofuran also showed better results when NiTPP was used instead of eosin [32–34] (Scheme 10).

Regarding protocols involving Ni complexes as catalysts, MacMillan and co-workers showed that Ni metallocatalysis can be successfully combined with photocatalysis (with Ir complexes) in a dual catalysis platform, which enables sp^3^–sp^3^ and sp^2^–sp^3^ bond formations [35]. In this context, metalloporphyrins emerge as an interesting platform for dual catalysis due to their ability to promote both metallocatalysis and photocatalysis in a one-pot system [36–39].

**Scheme 11:**
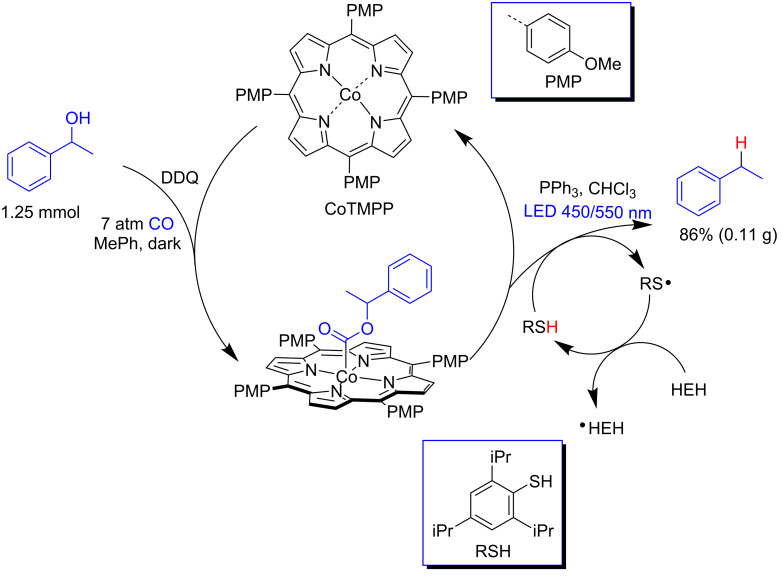
C–O bond cleavage of 1-phenylethanol using a cobalt porphyrin (CoTMPP) under visible light.

Martin and co-workers carried out the C–O bond cleavage of alcohols using a cobalt porphyrin under visible light irradiation and a carbon monoxide atmosphere (Scheme 11) [36]. The authors hypothesized that the C–O bond cleavage could be achieved via cobalt-mediated alcohol carbonylation followed by radical decarboxylation of the alkoxycarbonyl intermediate. In a proof-of-concept study, they proceeded with the carbonylation of 1-phenylethanol using Co(II) tetrakis(4-methoxyphenyl)porphyrin (CoTMPP) in the dark, furnishing the alkoxycarbonyl intermediate in 92% yield. The combination of this intermediate with a thiophenol derivative and Hantzsch ester (HEH) afforded ethylbenzene in 94% yield (86% overall yield) under both blue and green LED irradiation. The thiophenol and HEH were used as H donors for both the benzyl and thiyl radicals, respectively. The HEH was needed to avoid the dimerization of the thiophenol to the disulfide (Scheme 11).

**Scheme 12:**
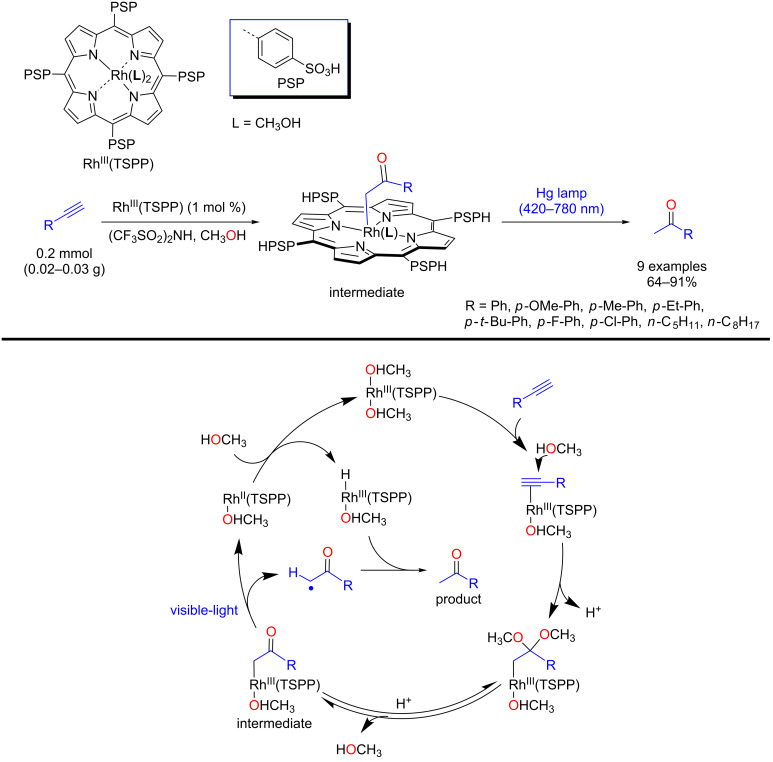
Hydration of terminal alkynes by Rh^III^(TSPP) under visible light irradiation.

Fu and co-workers have demonstrated the huge versatility of rhodium porphyrins for photocatalysis. In 2015, they showed that the hydration of terminal alkynes to ketones can be photocatalyzed by rhodium(III) tetrakis(*p*-sulfonylphenyl)porphyrin (Rh^III^TSPP) [37]. The coupling between Rh^III^(TSPP) and terminal alkynes produced the β-carbonylalkylrhodium porphyrin as a photoactive intermediate, whose irradiation produced the PhCOCH_2_ radical and Rh^II^(TSPP)(CH_3_OH) (Scheme 12). Then the Rh^II^(TSPP)(CH_3_OH) reacted with methanol to furnish H-Rh^III^(TSPP)(CH_3_OH) and Rh^III^(TSPP)(CH_3_OH)_2._ The ketones were produced in 64–91% yields after H transfer between H-Rh^III^(TSPP)(CH_3_OH) and the PhCOCH_2_ radical.

**Scheme 13:**
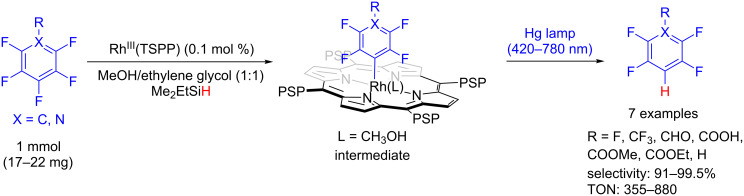
Regioselective photocatalytic hydro-defluorination of perfluoroarenes by Rh^III^(TSPP).

The same strategy was applied for the highly regioselective photoinduced hydro-defluorination of perfluoroarenes with Rh^III^(TSPP) [38]. The oxidative addition of the perfluoroarene to the metal complex furnished the active rhodium aryl complex intermediate, which led to the product after visible light irradiation. The hydro-defluorination products were obtained with good TON (up to 880) and high selectivity (91–99.5%), even though the aryl C–F bonds present a high bond dissociation energy (BDE) (Scheme 13).

**Scheme 14:**
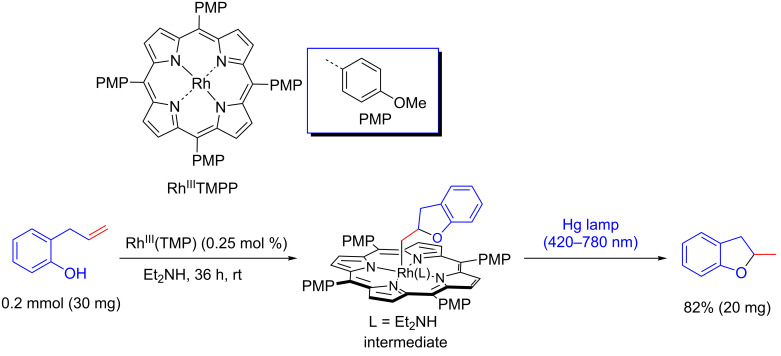
Formation of 2-methyl-2,3-dihydrobenzofuran by intramolecular hydro-functionalization of allylphenol using Rh^III^TMPP under visible light irradiation.

Using a similar photocatalytic system, 2-methyl-2,3-dihydrobenzofuran was produced by an intramolecular hydro-functionalization of *ortho*-allylphenol with rhodium(III) tetrakis(*p*-methoxyphenyl)porphyrin (Rh^III^TMPP) as photocatalyst (Scheme 14) [39]. The active intermediate (2,3-dihydrobenzofuran-2-yl)methyl rhodium porphyrin furnished the desired product in 82% yield.

**Scheme 15:**
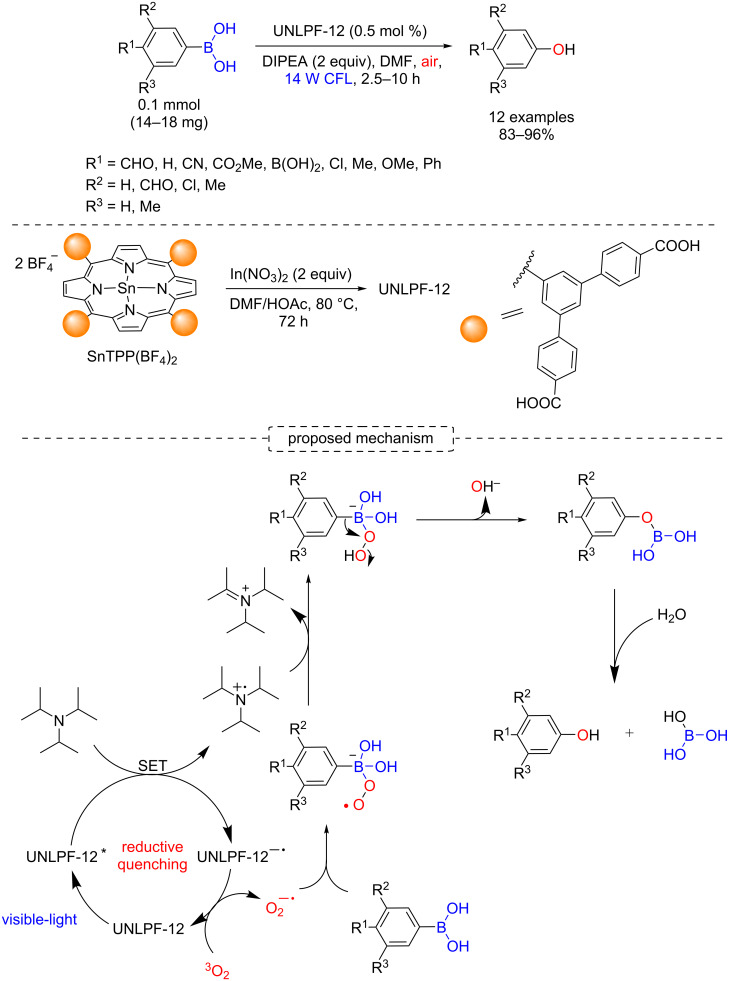
Photocatalytic oxidative hydroxylation of arylboronic acids using UNLPF-12 as heterogeneous photocatalyst.

Zhang and co-workers reported a protocol for an oxidative hydroxylation of arylboronic acids by a reductive quenching using a MOF Sn(IV) porphyrin-containing photocatalyst (UNLPF-12) under visible light irradiation. The authors obtained a variety of phenolic products in 83–96% yields (Scheme 15) [40].

The key steps of the mechanism are both the generation of superoxide radical anion by a reductive quenching and the rearrangement of the hydroperoxide intermediate [41]. The heterogeneous protocol using MOF porphyrins was significantly faster than the corresponding homogeneous photocatalysis, which was attributed to higher photostability of the porphyrins as MOF material. The UNLPF-12 presented practically the same photocatalytic efficiency even after the fourth recycle (from 99% to 95%), while the yield of the homogeneous photocatalysis dropped drastically in its second recycle (from 99% to 12%).

**Scheme 16:**
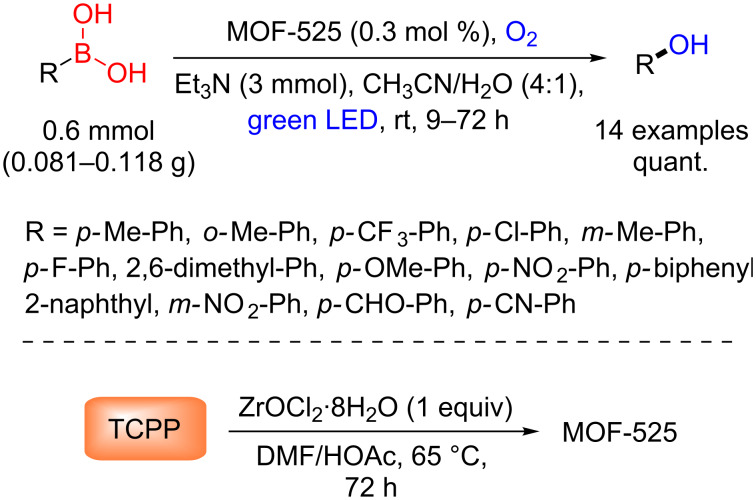
Photocatalytic oxidative hydroxylation of arylboronic acids using MOF-525 as heterogeneous photocatalyst.

In this regard, Horiuchi, Matsuoka and co-workers reported the synthesis of a Zr-based MOF with *meso*-tetrakis(4-carboxyphenyl)porphyrin (TCPP) (MOF-525, Zr_6_(OH)_4_O_4_(C_48_N_4_O_8_H_26_)_3_) [42] and showed its photocatalytic efficiency for oxidative hydroxylation of arylboronic acids [43]. The phenol products were obtained in quantitative yields for all evaluated arylboronic acids, but accompanied by long reaction times (Scheme 16).

**Scheme 17:**
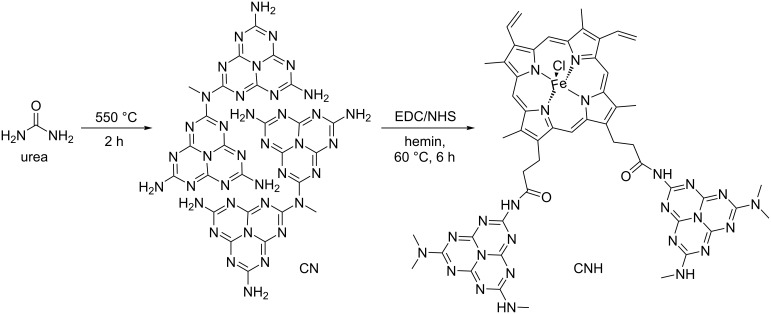
Preparation of the heterogeneous photocatalyst CNH.

In 2014, Yang, Huang, Wang and co-workers reported a photocatalytic sulfonation of alkenes to β-ketosulfones (widely used in the synthesis of compounds with biological activities [44]), using porphyrins supported in CN materials (Scheme 17) [45]. The thermal decomposition of urea at 550 °C for 2 h afforded the CN polymer, which possesses abundant –NH_2_ functional groups. The heterogeneous photocatalyst carbon nitride-hemin (CNH) was prepared after an amidation reaction between a carboxyl group of Fe(III) protoporphyrin IX and an amino group of the CN mediated by 1-(3-dimethylaminopropyl)-3-ethylcarbodiimide hydrochloride (EDC) and *N*-hydroxysuccinimide (NHS).

**Scheme 18:**
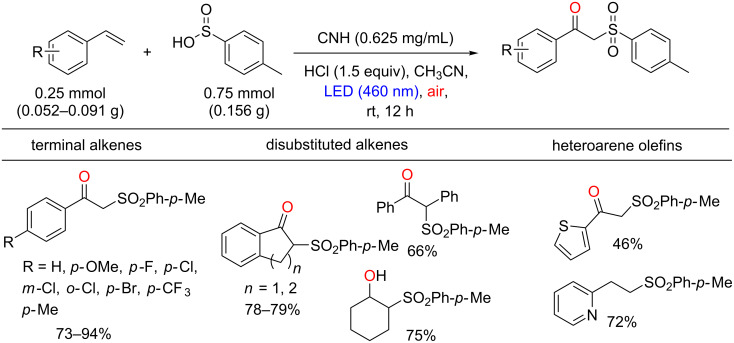
Photoinduced sulfonation of alkenes with sulfinic acid using CNH as photocatalyst.

The CNH was then applied as photocatalyst in the photoinduced sulfonation of styrene with *p*-methylbenzenesulfinic acid. The corresponding β-ketosulfone was obtained in 94% yield. However, the yield presented a slight decrease after 5 reuse cycles (from 94% to 85% yield). The methodology was applied to a variety of alkenes and the sulfinic acid, and the protocol was compatible with terminal, disubstituted and heteroarene alkenes (Scheme 18).

**Scheme 19:**
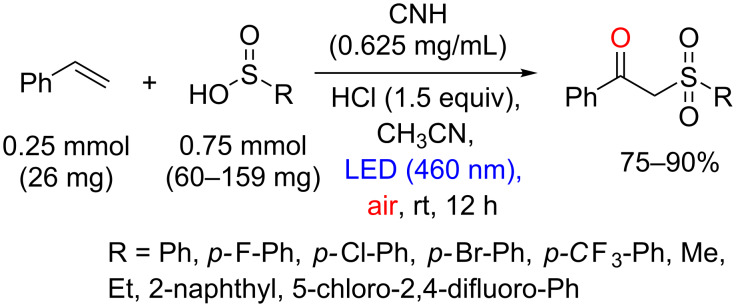
Sulfonic acid scope of the sulfonation reactions.

**Scheme 20:**
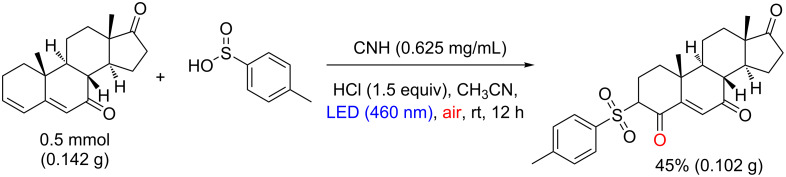
Regioselective sulfonation reaction of arimistane.

Relevant yields (up to 90%) were obtained for both electron-rich and electron-deficient groups in the arylsulfinic acid and alkyl sulfinates, such as sodium ethylsulfinate and sodium methylsulfinate (Scheme 19). The authors also showed that the methodology can be applied to the sulfonation of steroid drug arimistane in 45% yield (Scheme 20). The mechanistic studies on this reaction are ongoing with initial suggestions of the coupling between the sulfonyl radical and the radical alkene as a key step for this transformation.

**Scheme 21:**
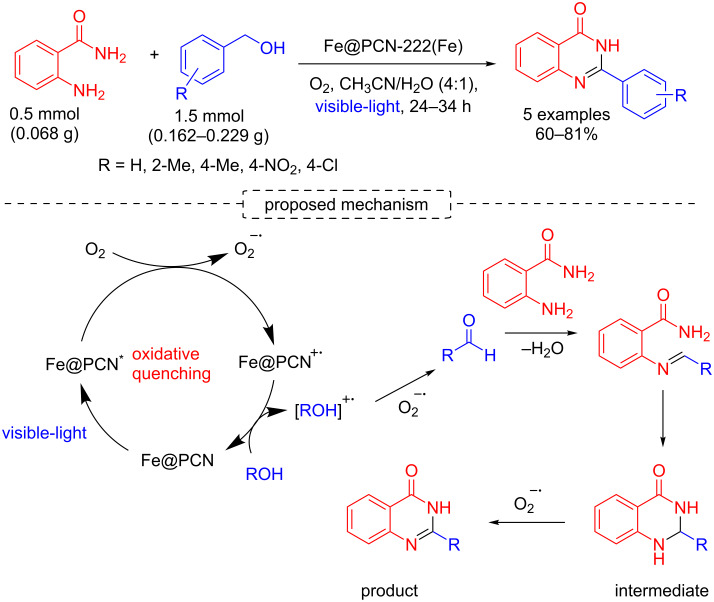
Synthesis of quinazolin-4-(3*H*)-ones.

In 2019, Ghaffari-Moghaddam, Oveisi and co-workers reported the synthesis of a new multifunctional MOF, namely Fe@PCN-222(Fe), and its application in the synthesis of quinazolin-4-(3*H*)-ones by the one-pot reaction between alcohols and 2-aminobenzamide under an oxidative quenching, visible light irradiation using air or oxygen as oxidant (60–81% yields). The authors propose the generation of the superoxide radical anion with the MOF. The superoxide radical anion oxidizes the alcohol to aldehyde, which is then converted to an imine after coupling with the amine. The products are obtained after cyclization and oxidation of the cyclic intermediates (Scheme 21) [46].

**Scheme 22:**
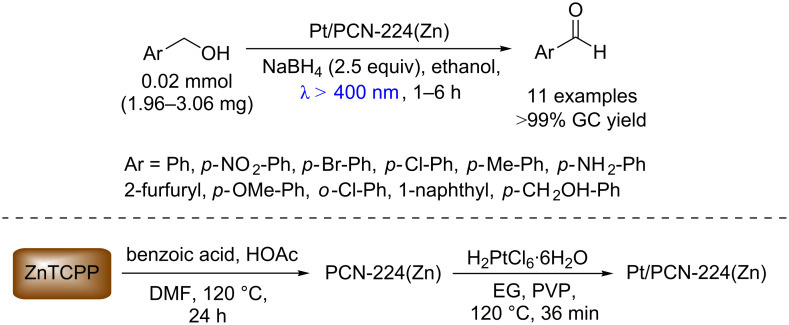
Selective photooxidation of aromatic benzyl alcohols to benzaldehydes using Pt/PCN-224(Zn).

Similarly, Jiang and co-workers reported the selective photooxidation of alcohols to aldehydes without over-oxidation to the carboxylic acids using Pt/PCN-224(Zn), a Zn porphyrin MOF incorporated with Pt nanocrystals (NC), as photocatalyst [47]. According to the authors, the synergism between NC and the porphyrin is important for this transformation and a wide variety of aldehydes were obtained with excellent conversions (>99%) using this heterogeneous photocatalyst (Scheme 22).

**Scheme 23:**
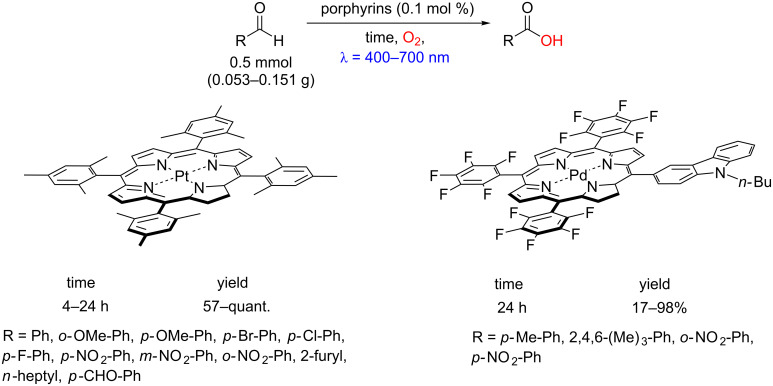
Photooxidation of benzaldehydes to benzoic acids using Pt or Pd porphyrins.

Metal complexes such as Pd and Pt porphyrins possess long-living triplet excited states and higher excited state potentials for oxidations [48]. In this regard, various benzoic acids were also obtained by photooxidation of benzaldehydes using Pt porphyrin (Pt-TMP) [49] and Pd porphyrin (2Pd) [50] (Scheme 23). Overall, the fine-tuning of the electrochemical potential of metals and porphyrins enables the oxidation of alcohols to aldehydes or aldehydes to carboxylic acids in a very controlled and chemoselective manner.

**Scheme 24:**
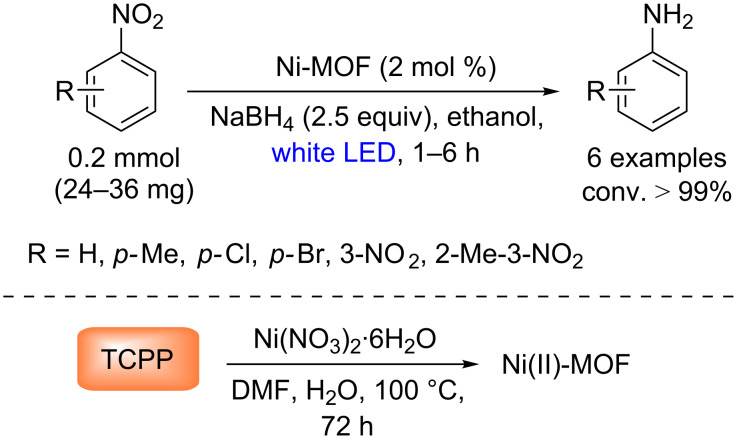
Photocatalytic reduction of various nitroaromatics using a Ni-MOF.

Regarding porphyrin-mediated reduction reactions, we have selected one representative example reported by Nagaraja and co-workers. They have shown a heterogeneous photocatalytic reduction of nitroaromatic compounds to the corresponding anilines using a Ni(II)-MOF, [Ni_3_(Ni-TCPP)_2_(µ_2_-H_2_O)_2_(H_2_O)_4_(DMF)_2_]·2DMF (where NiTCPP = Ni(II) *meso*-tetrakis(4-carboxyphenyl)porphyrin [51]) with excellent conversion (>99%) (Scheme 24). Surprisingly, they did not observe the target product when NiTCPP was used as photocatalyst.

**Scheme 25:**
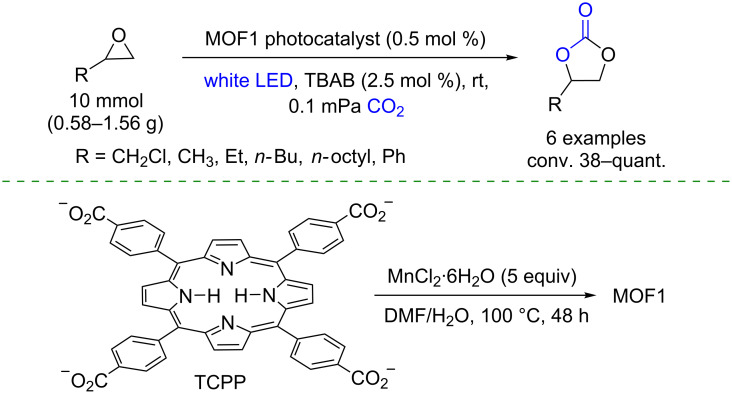
Photoinduced cycloadditions of CO_2_ with epoxides by MOF1.

Finally, it has been found that heterogeneous porphyrin-based photocatalysis also can be applied to the reduction of carbon dioxide (CO_2_). Many photocatalytic materials containing porphyrins have been developed for CO_2_ reactions in organic synthesis, including photoinduced transformations [52–55]. Nagaraja and co-workers reported the first porphyrin-based MOF for photoinduced cycloaddition of carbon dioxide with epoxides [56]. The synthesis of a 3D supramolecular framework, [{Mn(TCPP)_0.5_(H_2_O)}·2H_2_O]*_n_*, where Mn(TCPP) is Mn(II) *meso*-tetrakis(4-carboxyphenyl)porphyrin, was derived from MnCl_2_·6H_2_O and TCPP. The cycloaddition product, a cyclic carbonate, was obtained from different epoxides, whose conversion was proportional to the substrate size, using MOF1 and tetra-*n*-butylammonium bromide (TBAB) as photocatalyst and co-catalyst, respectively (Scheme 25). A successful gram-scale protocol was developed opening up perspectives for using these materials in CO_2_ reuse.

### Porphyrins as energy transfer photocatalysts

#### General aspects

As previously mentioned, porphyrins in their excited state can also return to the ground state by energy transfer. In this section, we highlight the process of energy transfer from the triplet excited state of porphyrins to the steady state of molecular oxygen (triplet state). In this process, well-known singlet oxygen (^1^O_2_) is generated.

Singlet oxygen can be considered a very versatile reagent in organic synthesis since it promotes many mild oxidation processes instead of combustion [23,57–60]. This excited state form of molecular oxygen can be produced by chemical and photochemical methods, with this second the easier and most cost-competitive manner [61–62].

**Figure 4:**
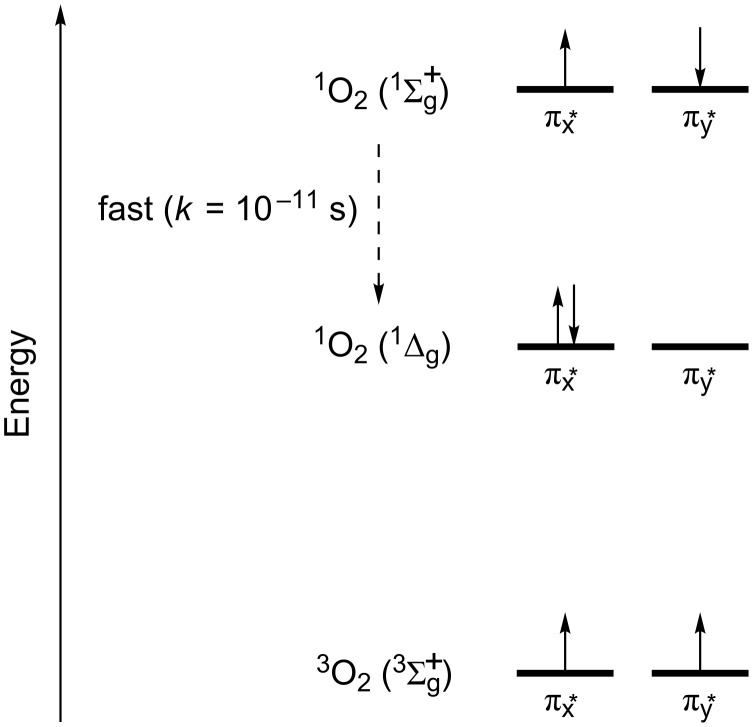
Electronic configurations of the species of oxygen. Adapted from [66].

Singlet oxygen exists in a very unique electronic configuration relative to other molecules, and presents two singlet states, ^1^O_2_(^1^Δ_g_) and ^1^O_2_(^1^Σ_g_^+^) (Figure 4) [63]. The species ^1^O_2_(^1^Δ_g_) has both electrons paired in a single orbital, while the electrons of the species ^1^O_2_(^1^Σ_g_^+^) are paired in different orbitals. Both singlet states can be formed competitively, however, the conversion from ^1^O_2_(^1^Σ_g_^+^) to ^1^O_2_(^1^Δ_g_) is extremely fast in liquid-phase (*k* = 10^−11^ s), making the ^1^O_2_(^1^Δ_g_) the main photoactive species in solution [63–65].

**Scheme 26:**
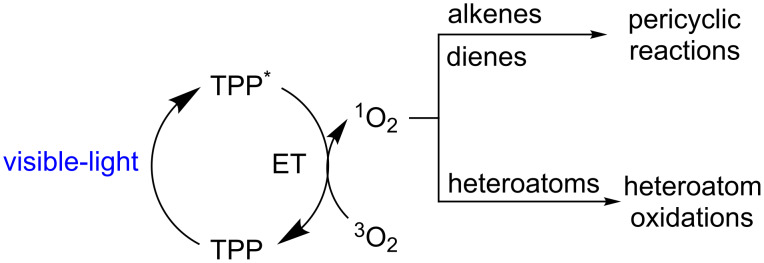
TPP-photocatalyzed generation of ^1^O_2_ and its application in organic synthesis. Adapted from [67–69].

Singlet oxygen is a highly reactive electrophile toward electron-rich organic molecules/atoms such as alkenes, dienes, and heteroatoms (N, P, S, Se, etc.) making this molecule very effective in pericyclic reactions and heteroatom oxidations (Scheme 26) [61,67–68]. In this section, both reactions are presented and discussed.

#### Singlet oxygen in pericyclic reactions

**Scheme 27:**
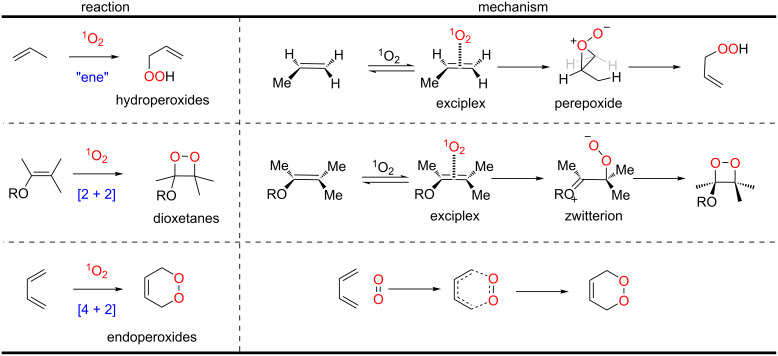
Pericyclic reactions involving singlet oxygen and their mechanisms. Adapted from [67].

Many important organic transformations can be performed by singlet oxygen including ene, [2 + 2] and [4 + 2] cycloaddition reactions for the formation of hydroperoxides, dioxetanes, and endoperoxides, respectively (Scheme 27). The mechanistic foundations that allow the predictability and the rational use of these reactions in organic synthesis are well-established. However, studies about the pathways in which these pericyclic reactions with singlet oxygen occur (stepwise or concerted) are still ongoing. The generally accepted mechanisms for these reactions are shown in Scheme 27, and propose a stepwise mechanism for ene and [2 + 2] cycloaddition, and a concerted mechanism for [4 + 2] cycloaddition [67].

In this part of the review, we decided to highlight the historically relevant protocols and recent reactions involving ^1^O_2_, mostly developed with porphyrin photocatalysis. No examples of the use of chlorins, bacteriochlorins and isobacteriochlorins will be included, except two seminal examples of chlorophyll a use (formally, chlorophyll a is a chlorin derivative). However, for a broad coverage of different aspects and relevant examples on photooxygenation in organic synthesis, we recommend relevant reviews published by Greer [70] and Crutchley [71].

**Scheme 28:**
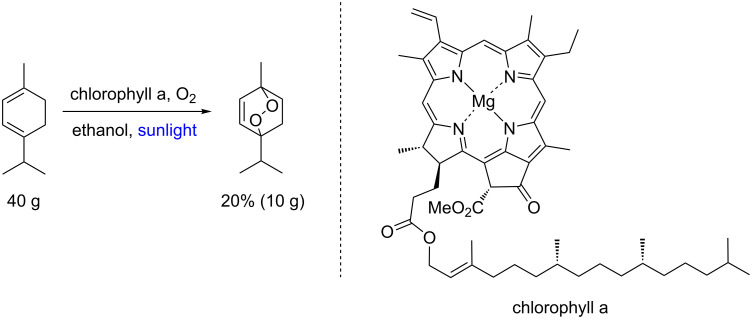
First scaled up ascaridole preparation from α-terpinene.

Undoubtedly, the large-scale preparation of ascaridole, published in 1944 by Schenck and Ziegler [72–73], is a remarkable example of the use of chlorophyll a in photooxygenation reactions (Scheme 28). In this seminal scaled-up preparation the authors were able to produce 10 g of ascaridole per batch, showing an additional example of batch numbering up of photoreactors with dozens of glass batch systems scattered in an open garden under sunlight irradiation; notably, ascaridole is an effective anthelmintic natural drug obtained in this protocol from another readily available natural product α-terpinene.

**Scheme 29:**
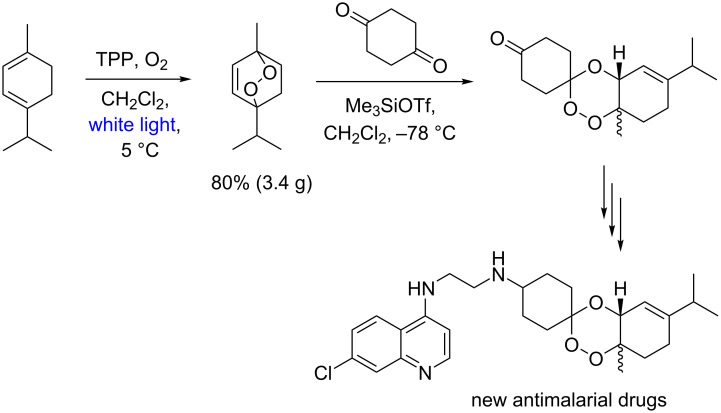
Antimalarial drug synthesis using an endoperoxidation approach.

Several other approaches have been described in both synthesis and derivatizations of ascaridole. However, the Meunier group publication [74] on new antimalarial drugs can be highlighted (Scheme 29). The authors report a 3.4 gram-scale preparation of ascaridole, in this case a synthetic intermediate, using TPP as photocatalyst.

**Scheme 30:**
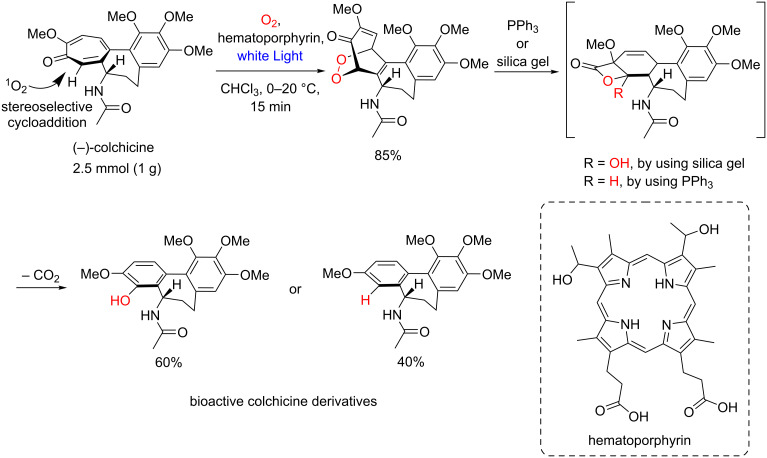
Photooxygenation of colchicine.

Another example of a scaled-up endoperoxide approach involves the stereoselective singlet oxygen addition to (−)-colchicine photocatalyzed by hematoporphyrin, giving relevant bioactive colchicine derivatives (Scheme 30) [75–76].

**Scheme 31:**
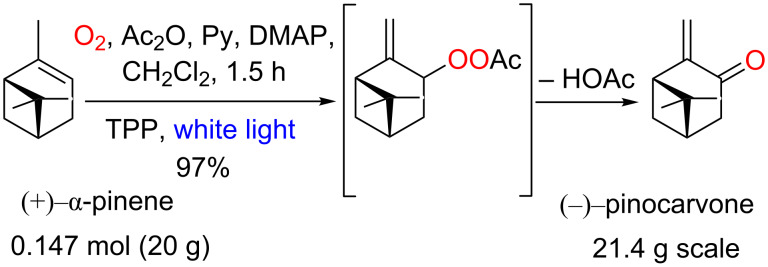
Synthesis of (−)-pinocarvone from abundant (+)-α-pinene.

A valuable protocol for the (−)-pinocarvone synthesis was described by Eickhoff [77] and recently adapted to continuous-flow conditions by Lapkin and co-workers [78]. In the original protocol the authors were able to produce up to 21.4 g per batch (1.5 h, 97% yield) using TPP as photocatalyst (Scheme 31). In the recent continuous protocol described by Lapkin et al. 1.9 g per day is obtained using the same reaction conditions and a PFA-tube photoreactor (segmented-flow with O_2_).

**Scheme 32:**
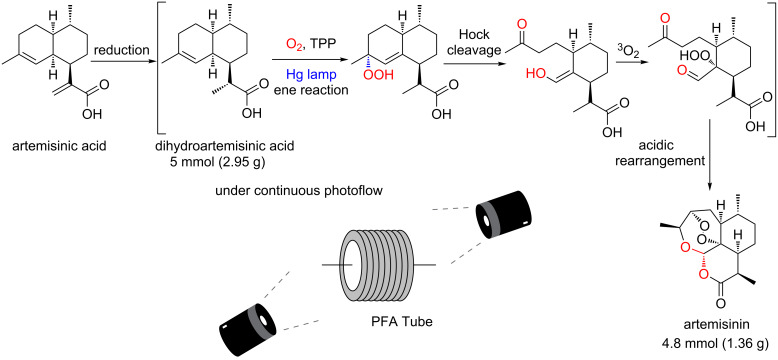
Seeberger’s semi-synthesis of artemisinin.

One very relevant example of a photooxygenation protocol is the Seeberger group’s semi-synthesis of the antimalarial API artemisinin (Scheme 32) [79]. They have established a gram-scale continuous protocol with a photooxygenation promoted by TPP starting from dihydroartemisinic acid**.** This work is crucial for the success of the subsequent industrial process introduced by the pharmaceutical company Sanofi [80].

**Scheme 33:**
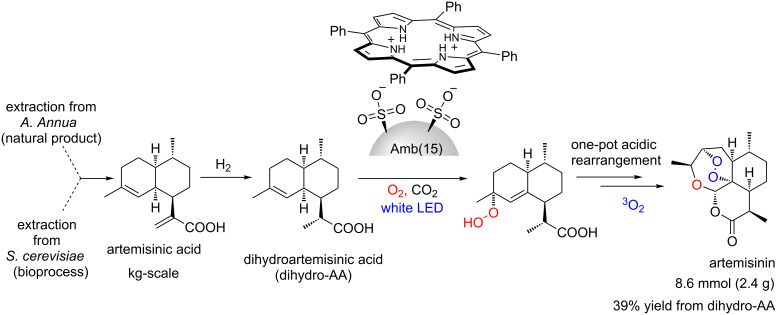
Synthesis of artemisinin using TPP and supercritical CO_2_.

After this seminal publication, a consortium between Sanofi and UK/China universities also reported a green protocol for artemisinin synthesis using supercritical CO_2_ as a novelty (Scheme 33) [81]. They have also used TPP (immobilized acidic form) as photosensitizer and were able to produce up to 2.4 g of artemisinin per batch. The importance of artemisinin for Big Pharma has been confirmed during the last 20–30 years with many relevant publications.

**Scheme 34:**
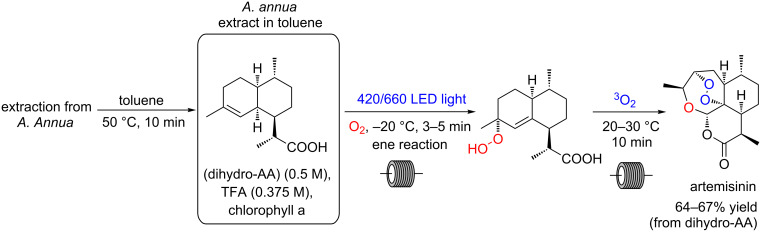
Synthesis of artemisinin using chlorophyll a.

As mentioned before, our focus in this part of the review is the use of porphyrins as photocatalysts, but we decided to select another relevant example using chlorophyll a (a chlorin-type derivative) for artemisinin preparation. Gilmore and co-workers have shown that the extract in toluene from *Artemisia annua* plants contains both the substrate (dihydroartemisinic acid) and the photocatalyst (chlorophyll a). The combination of the extract with trifluoroacetic acid promptly furnishes artemisinin, under continuous-flow conditions (3 min) using red light (87% yield) or under blue light irradiation (5 min, 88% yield) (Scheme 34) [82].

The most recent approach for artemisinin synthesis was described by Wang, Zhou and co-workers [83–84] using porphyrin-based MOFs to produce singlet oxygen, and then repeating the same synthetic strategy for artemisinin production (starting from artemisinic acid).

**Scheme 35:**
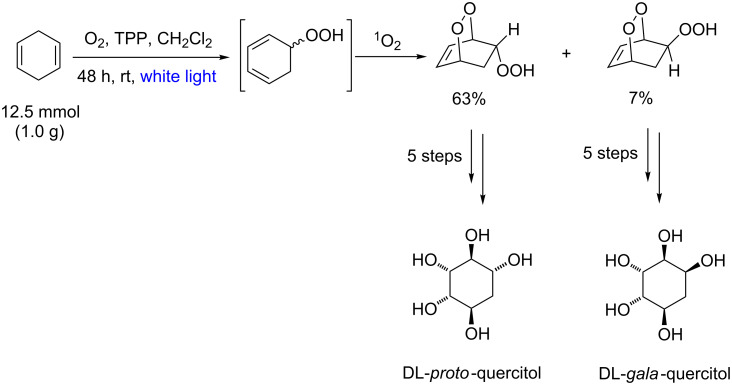
Quercitol stereoisomer preparation.

Photooxygenation reactions were also described in the syntheses of carbasugars which are unnatural molecular motifs with broad interest in medicine [85]. A relevant example was described by Balci and co-workers for the preparation of quercitol derivatives (Scheme 35) [86]. In this case, gram to multigram-scale double photooxygenation reactions were described using TPP as photocatalyst.

**Scheme 36:**
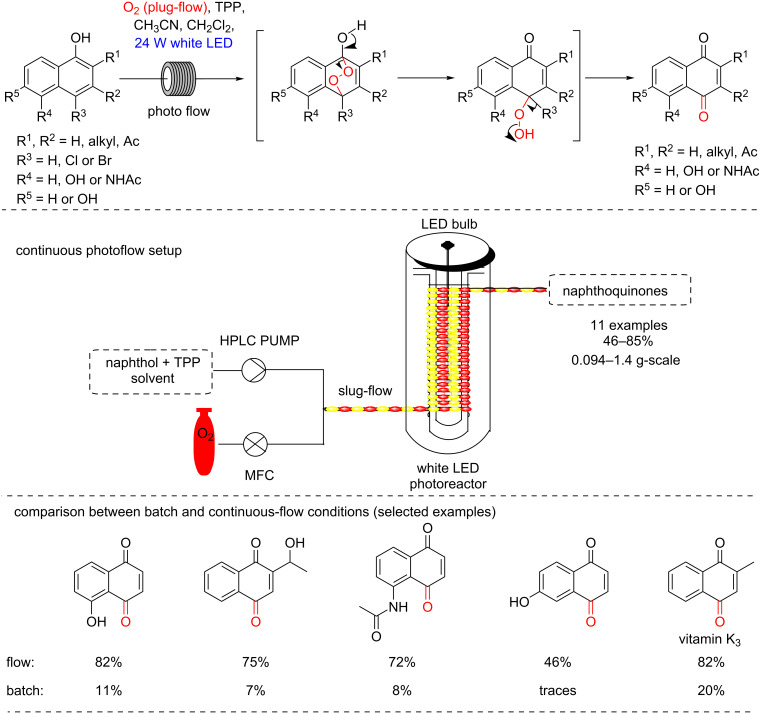
Photocatalyzed preparation of naphthoquinones.

We have described a study on endoperoxydations followed by rearrangement to yield naphthoquinones starting from α-naphthols and using porphyrins as photocatalysts (Scheme 36) [23]. Eleven examples were described from mg to g-scale reactions, and including protocols with 24 h experiments under continuous-flow conditions using a very simple home-made photoreactor (segmented flow – PFA tube reactor). We have compared the same reaction conditions in both batch (7–20% yield) and continuous-flow conditions (up to 82% yield) and thus showed a very improved protocol when using continuous conditions.

**Scheme 37:**
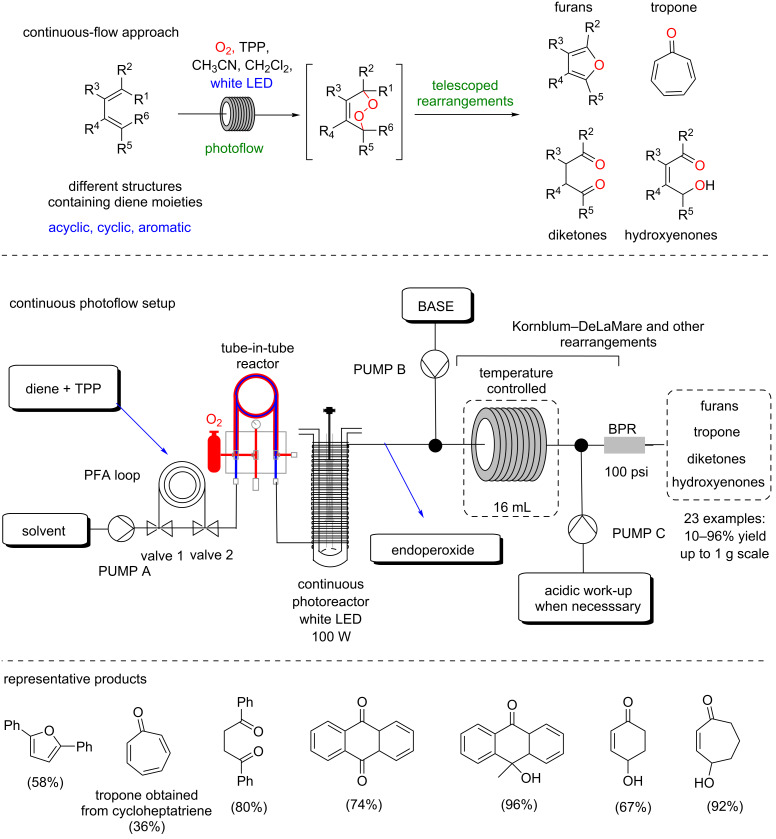
Continuous endoperoxidation of conjugated dienes and subsequent rearrangements leading to oxidized products.

Subsequently, we reported a comprehensive methodology involving photooxygenations of conjugated dienes and rearrangements, thus leading to relevant oxidized products (Scheme 37) [87]. In this methodology, we developed in both batch and continuous-flow conditions, a porphyrin-based protocol for endoperoxidation of the diene, followed by the Kornblum–DeLaMare rearrangement and further telescoped transformations. This protocol yields different classes of products such as furans, tropone, diketones and hydroxyenones, all of them starting from the corresponding functionalized dienes. A scope with 23 substrates is presented and the products were obtained in 10–96% yield with scalability (up to 1 g-scale in a telescoped protocol).

**Scheme 38:**
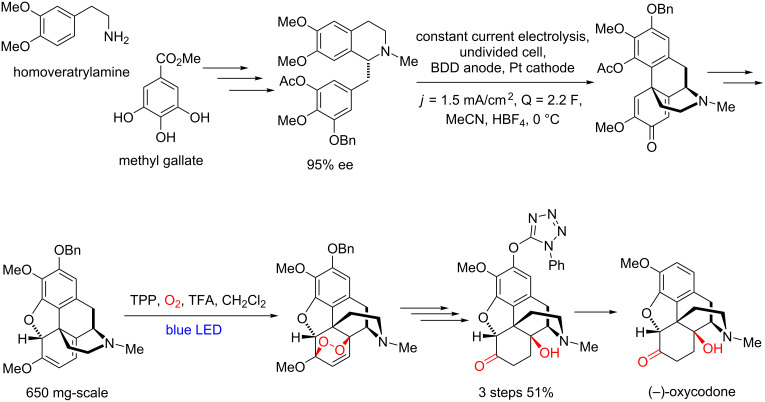
The Opatz group total synthesis of (–)-oxycodone.

In 2019, Opatz and co-workers reported one of the most efficient and elegant total syntheses of (–)-oxycodone, using as key steps an electrochemical cyclization and an endoperoxidation photocatalyzed by TPP in an almost gram-scale (Scheme 38) [88].

**Scheme 39:**
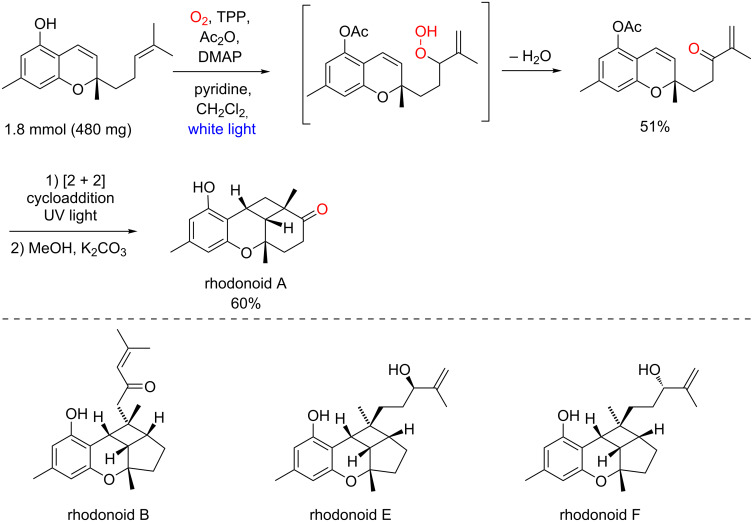
Biomimetic syntheses of rhodonoids A, B, E, and F.

In 2020, Burchill and George reported a 0.5 g-scale ene-reaction with singlet oxygen and a cromene derivative, thus giving a conjugated enone after a Kornblum–DeLaMare rearrangement (Scheme 39) [89]. Further photochemical [2 + 2] cycloaddition and hydrolysis allowed them to obtain rhodonoid A in 30% overall yield. Other similar natural products (rhodonoid B, E and F) were also prepared by the same synthetic strategy.

**Scheme 40:**
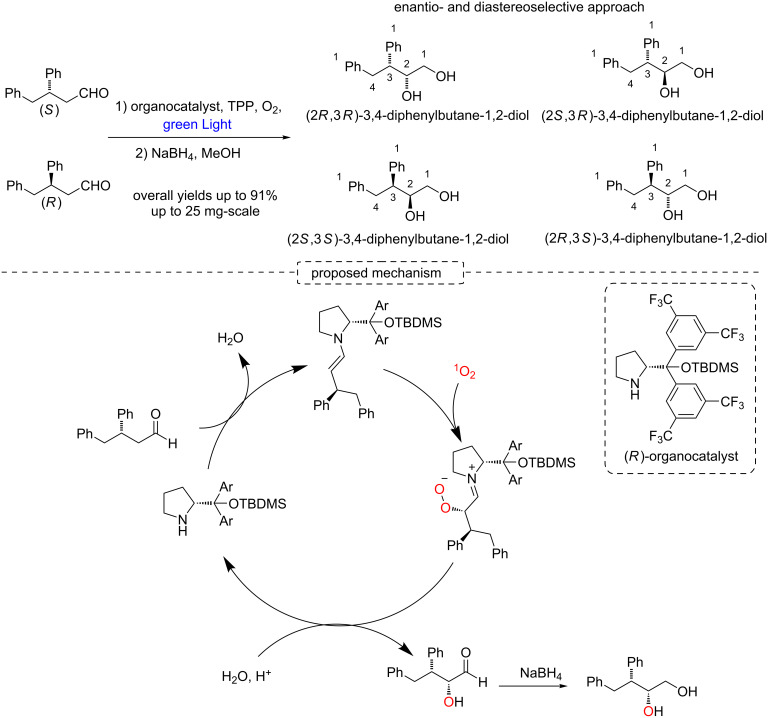
α-Photooxygenation of chiral aldehydes.

Singlet oxygen has also been efficiently used for enantioselective and chemoselective oxidations of many organic compounds. Notably, the Gryko’s group recently described an enantio- and diastereoselective approach involving a porphyrin-based photooxygenation of aldehydes with sequential reduction to yield chiral diols in yields up to 91% and significant er (up to 96:4), but low dr (up to 66:33) (Scheme 40) [90].

**Scheme 41:**
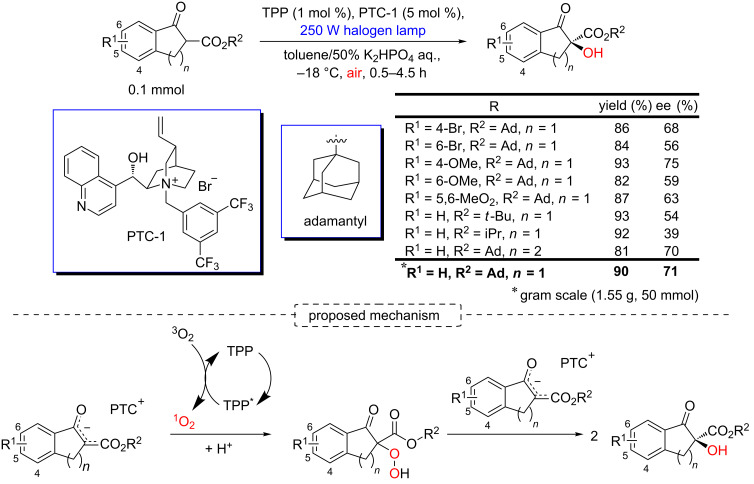
Asymmetric photooxidation of indanone β-keto esters by singlet oxygen using PTC as a chiral inducer, and the related mechanism.

Another relevant example has been described by Meng and co-workers with the synthesis of α-hydroxy-β-keto esters using TPP, a visible-light source, and a phase-transfer catalyst (PTC) as enantio-catalyst [91]. They reported the preparation of indanone-α-hydroxy-β-keto esters in 81–93% yields and 39–75% ee (Scheme 41). The mechanism of this reaction involves the attack of the enolate paired with the chiral counter ion PTC to the singlet oxygen electrophile to give the hydroperoxide intermediate, which is converted to α-hydroxy-β-keto esters (Scheme 41) [91].

**Scheme 42:**
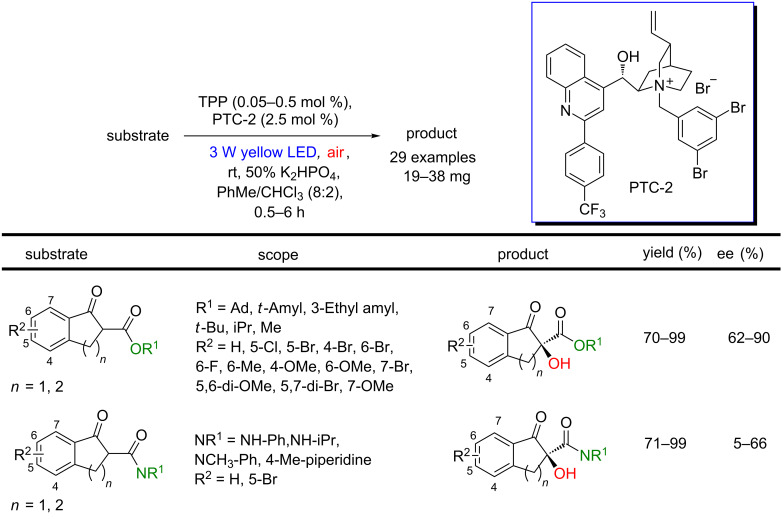
Asymmetric photooxidation of both β-keto esters and β-keto amides by singlet oxygen using PTC-2 as a chiral organocatalyst.

Later, these results were improved by the development of a new chiral PTC and re-optimization of the experimental conditions [92]. The new protocol furnished the indanone derivatives in 70–99% yields and 62–90% ee. Furthermore, the methodology was also applied to oxidations of β-keto amides (71–99% yields) and with 5–66% ee (Scheme 42).

**Scheme 43:**
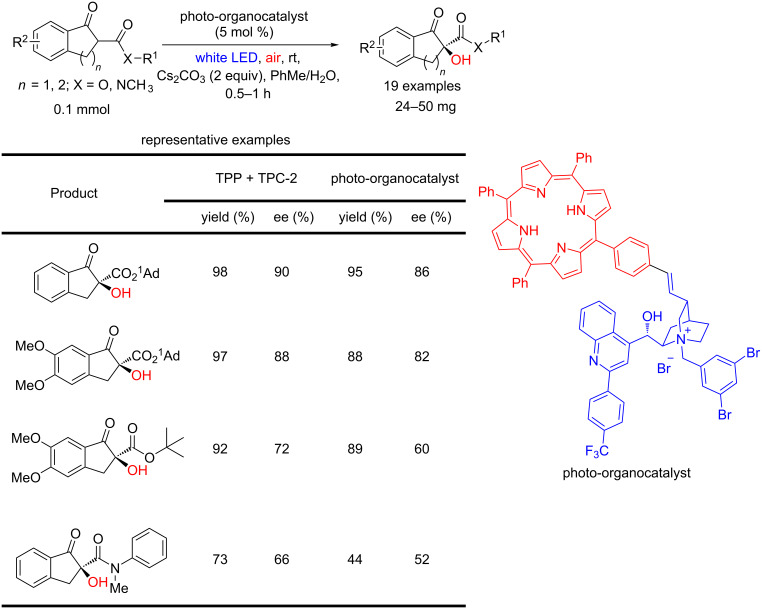
Bifunctional photo-organocatalyst used for the asymmetric oxidation of β-keto esters and β-keto amides by singlet oxygen.

In 2018, Meng and co-workers developed a bifunctional photo-organocatalyst combining both the photosensitizer and the chirality inducer. Relevant enantiomeric excesses were observed (up to 86% ee) in the oxidation of both β-keto esters and β-keto amides (Scheme 43) [93].

#### Singlet oxygen in heteroatom oxidations

Singlet oxygen reacts readily with electron pairs of heteroatoms, such as sulfur, selenium, phosphorus, and nitrogen, due to their electrophilicity. The interaction between singlet oxygen and the heteroatom occurs in both physical and chemical quenching leading to the formation of covalent products [67].

**Sulfur oxidation:** One of the first examples reported for heteroatom oxidation by singlet oxygen was the oxidation of sulfides to sulfoxides [67]. Sulfoxides are important intermediates in organic synthesis, and with applications in medicine and pharmacology [94], justifying many studies on this topic.

**Scheme 44:**
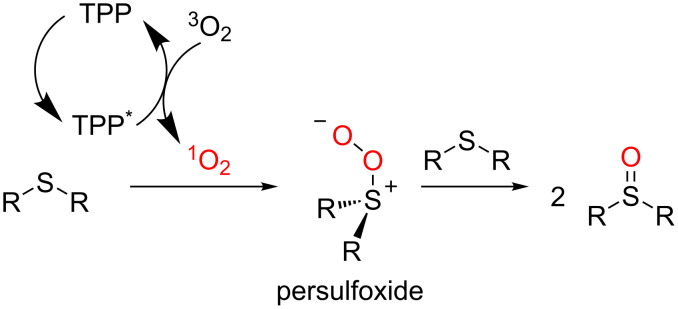
Mechanism of singlet oxygen oxidation of sulfides to sulfoxides.

The accepted mechanism for sulfide oxidation to sulfoxide involves the chemical quenching of singlet oxygen by sulfur compounds which leads to the persulfoxide intermediate. From this intermediate, a variety of reaction pathways are suggested to give the oxidized product, including the quenching with a second molecule of sulfide (Scheme 44) [67,95–96].

**Scheme 45:**
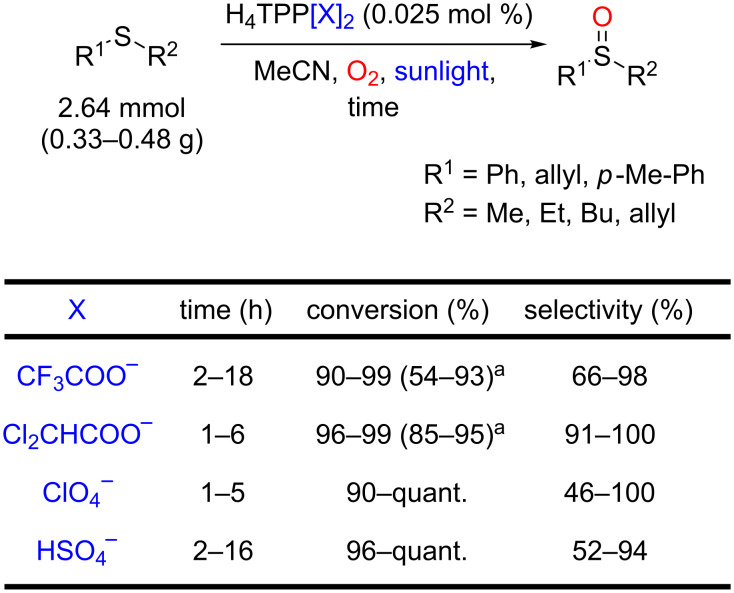
Controlled oxidation of sulfides to sulfoxides using protonated porphyrins as photocatalysts. ^a^Isolated yield.

Recent advances have also been achieved using photostable porphyrins and/or heterogeneous catalysts. Mojarrad and Zakavi reported that the oxidation of sulfides using diprotonated porphyrins as photocatalysts under sunlight irradiation furnished the corresponding sulfoxides with high chemoselectivity (up to 100%), scalability (up to 2.6 mmol) and high yields (up to 100%) [94]. According to the authors, the protonation of the porphyrins causes a red-shift of the photosensitizer with an increase of singlet oxygen generation and photocatalytic activity under the sunlight irradiation. Furthermore, the steric hindrance around the porphyrin core, caused by the diacids, enhanced the catalyst photostability, allowing a lowers porphyrin load. The authors evaluated various protonated-TPPs, using CF_3_COOH, Cl_2_CHCOOH, HClO_4_ and H_2_SO_4_ acids, and they concluded that H_4_TPP(ClO_4_)_2_ and H_4_TPP(Cl_2_CHCOO)_2_ were the most stable and efficient photocatalysts of the series (Scheme 45).

**Scheme 46:**
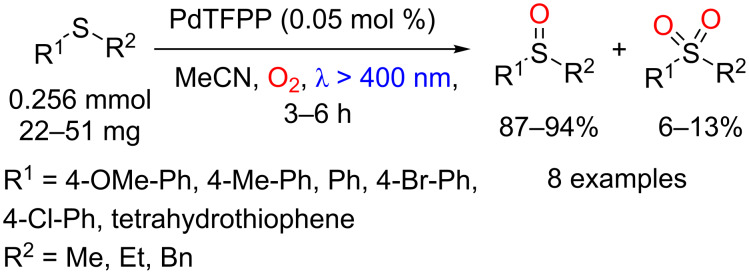
Photochemical oxidation of sulfides to sulfoxides using PdTPFPP as photocatalyst.

Che and co-workers showed that Pd(II) *meso*-tetrakis(pentafluorophenyl)porphyrin (PdTPFPP) can be used for the conversion of sulfides to sulfoxides via oxidation by singlet oxygen [97]. A series of sulfides was oxidized to the corresponding sulfoxides in 87–94% yields using only 0.05 mol % of the photocatalyst (TON: 1880) (Scheme 46).

**Scheme 47:**
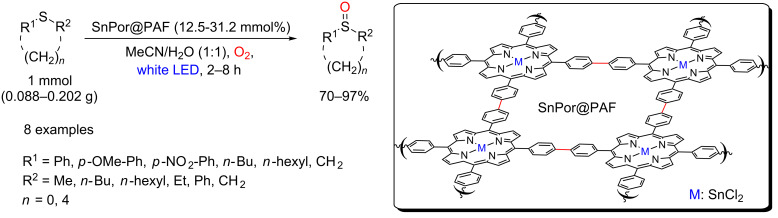
Controlled oxidation of sulfides to sulfoxides using SnPor@PAF as a photosensitizer.

The controlled oxidation of sulfides to sulfoxides by singlet oxygen was also reported using heterogeneous photocatalysts. A Sn porphyrin-based porous aromatic framework (SnPor@PAF) with a broad and strong optical absorption in the visible light region was used for this transformation [98]. Luo, Ji and co-workers synthesized this material by a Yamamoto homo coupling reaction using a well-designed brominated tin porphyrin (SnTBPP) as monomer (Scheme 47). The irradiation of this material in the presence of both sulfides and molecular oxygen furnished a variety of sulfoxides in 70–97% yields. The SnPor@PAF presented the same photocatalytic activity of its monomer (SnTBPP) with the advantage of its easy recovery and reuse. The authors did not observe any decrease in the photocatalytic activity of the material even after four reuses.

**Scheme 48:**
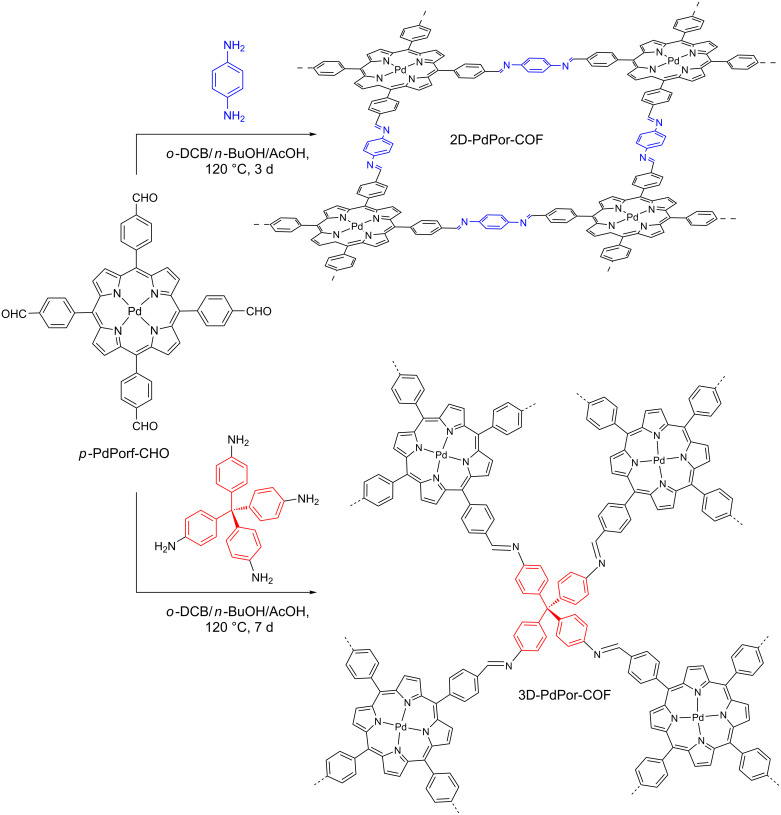
Syntheses of 2D-PdPor-COF and 3D-Pd-COF.

Another very promising catalytic platform for this transformation is the covalent organic framework (COF), a class of porous crystalline polymers built from molecular building blocks linked via covalent bonds. Recently, Sun, Wang and co-workers built both 2D- and 3D-porphyrin COFs (2D-PdPor-COF and 3D-PdPor-COF, respectively) from the same porphyrin, Pd(II) *meso*-tetrakis(4-formylphenyl)porphyrin (*p*-PdPor-CHO) (Scheme 48) [99]. In the 2D-COF, the functional moieties in the adjacent layers have strong π–π interactions that could be beneficial for the charge mobility. On the other hand, the three-dimensionally organized (3D-COF) allows open sites. Therefore, for the first time, the photocatalytic activity of the same porphyrin was evaluated in distinct dimensional frameworks.

**Scheme 49:**
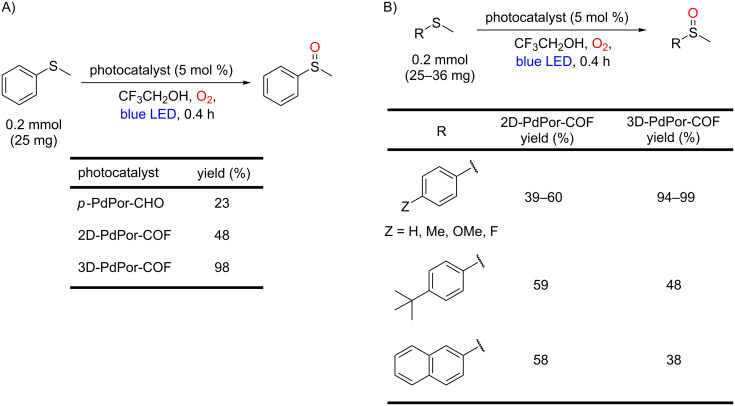
Photocatalytic oxidation of A) thioanisole to methyl phenyl sulfoxide and B) various aryl sulfides, using 2D-PdPor-COF, 3D-PdPor-COF, and *p*-PdPor-CHO.

The authors showed that the photocatalytic performance of COF-porphyrin (2D-COF = 48% and 3D-COF = 98%) was significantly higher than in the case of homogeneous photocatalysis (*p*-PdPor-CHO = 23%) (Scheme 49A). Among COF-porphyrins, the 3D-COF presented the highest activity for all smaller substrates, such as the *p*-Me-Ph substituent (99%), but lower activity for the bigger naphthyl substrate (38%). The achieved yields for 2D-COF were moderate for all the evaluated substrates (39–60%) (Scheme 49B). Thus, the authors suggested that the 3D-COF, whose porous size (0.63 nm) is smaller than the 2D-COF (1.87 nm), acts as a size-selective photocatalyst. Furthermore, the photocatalytic activity of 2D-COF is lower due to the π–π interaction between framework layers.

**Scheme 50:**
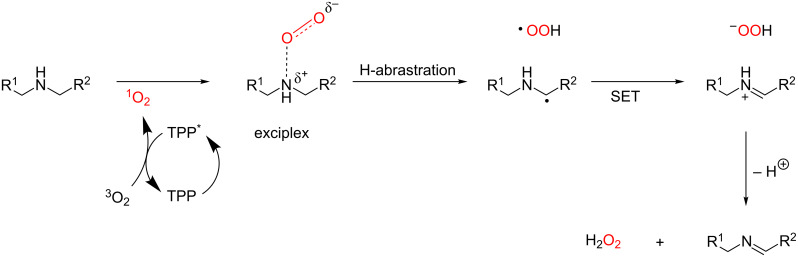
General mechanism for oxidation of amines to imines.

**Nitrogen oxidation:** Amines are well-known as very efficient physical and chemical quenchers for singlet oxygen [67,100–101]. A myriad of chemical transformations come from this process, whose crucial step involves the formation of a charge-transfer complex between singlet oxygen and the amine. Subsequently, hydrogen-atom abstraction leads to the radical intermediate, which can undergo SET with a hydroperoxyl radical to afforded an iminium ion, then giving an imine after deprotonation (Scheme 50).

**Scheme 51:**
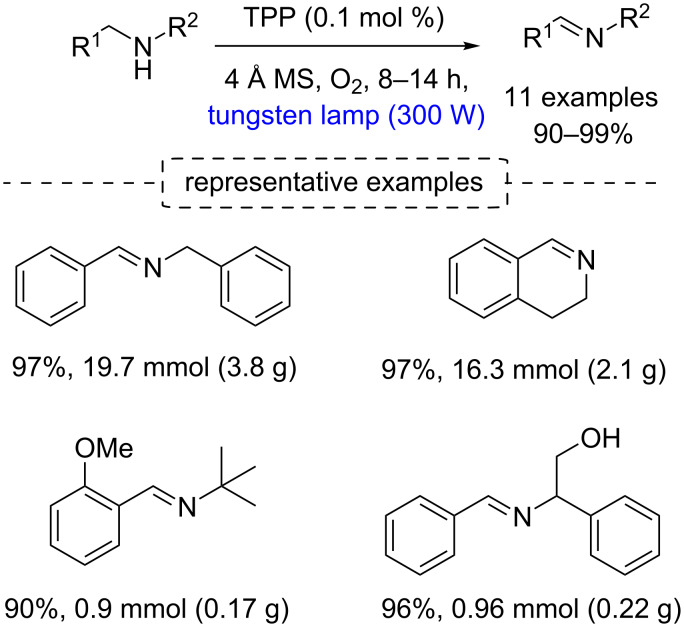
Oxidation of secondary amines to imines.

Adopting this strategy, Che and co-workers obtained several imines in 90–99% yield from secondary amines [102] (Scheme 51). The authors observed that the oxidation is regioselective, occurring at the less substituted position of nonsymmetric dibenzylamines, and they have demonstrated scalability of the protocol (products in up to 3.8 g scale).

**Scheme 52:**
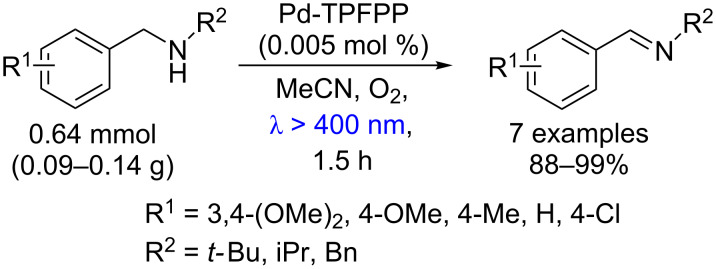
Oxidation of secondary amines using Pd-TPFPP as photocatalyst.

Similarly, using Pd-TPFPP the oxidation of amines to imines was achieved in 88–99% yields with low loading (0.005 mol %, 13000 TON h^−1^) after 1.5 h of irradiation with visible light (Scheme 52) [97].

The oxidation of amines to imines was also described using heterogeneous catalysis.

**Scheme 53:**
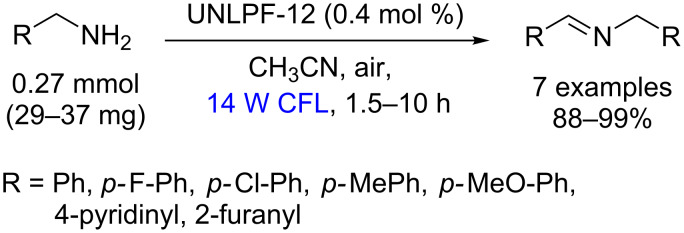
Oxidative amine coupling using UNLPF-12 as heterogeneous photocatalyst.

Zhang and co-workers demonstrated that the MOF (Sn^IV^)porphyrin-containing photocatalyst (UNLPF-12) can be used for the oxidation of primary amines to imines in 88–99% yields under visible light irradiation (Scheme 53) [40]. In this case, the authors observed the oxidative coupling between the primary amines and their respective imines to produce the secondary imines.

**Scheme 54:**
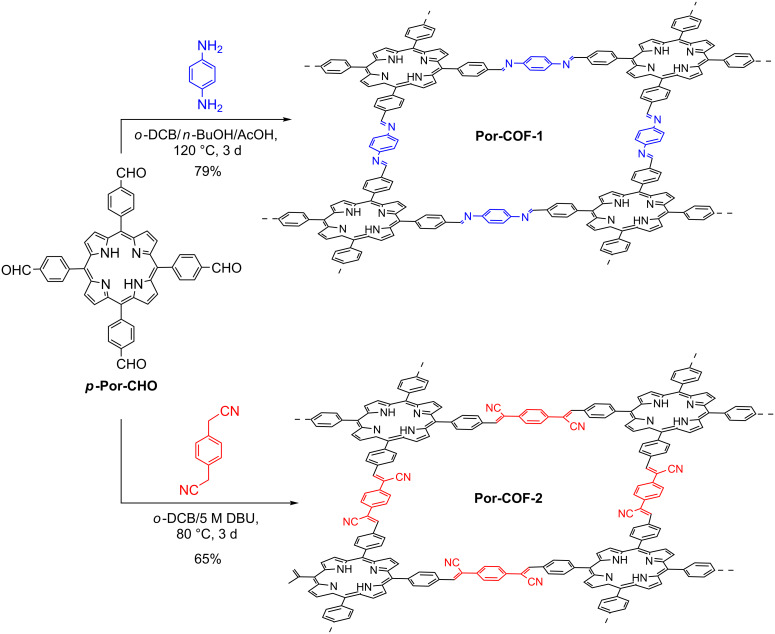
Synthesis of Por-COF-1 and Por-COF-2.

Wang and co-workers reported the synthesis, characterization, and application of interesting metal-free heterogeneous photocatalysts, 2D porphyrin-COFs (Por-COF), which were obtained by condensation between *meso*-tetrakis(4-formylphenyl)porphyrin (*p*-Por-CHO) and benzene-1,4-diamine, and 1,4-phenylenediacetonitrile, for Por-COF-1 and Por-COF-2, respectively (Scheme 54) [103]. The sp^2^ carbon-linked COF conferred high chemical stability to the material (Por-COF-2) due to the low reversibility of the double bond formation.

**Scheme 55:**
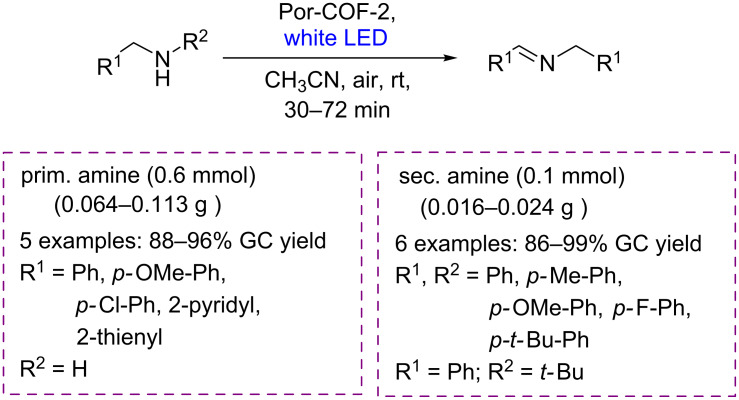
Photocatalytic oxidation of amines to imines by Por-COF-2.

Initially, the photocatalytic oxidative amine coupling was selected as a reaction model [103]. The authors observed that the imine-based Por-COF-1, decomposed completely, and no target product was detected. Nevertheless, the Por-COF-2 presented high photocatalytic activity for this transformation. The *N*-benzylidenebenzylamines were obtained in excellent yields (86–99%) for primary and secondary amine derivatives bearing electron-donating and electron-withdrawing groups (Scheme 55).

Imines are useful building blocks for the synthesis of biologically active compounds [104]. These compounds can be trapped with nucleophiles to produce the α-amino-substituted compounds, and be employed as substrates for a variety of chemical transformations such as Ugi- and Mannich-type reactions.

**Scheme 56:**
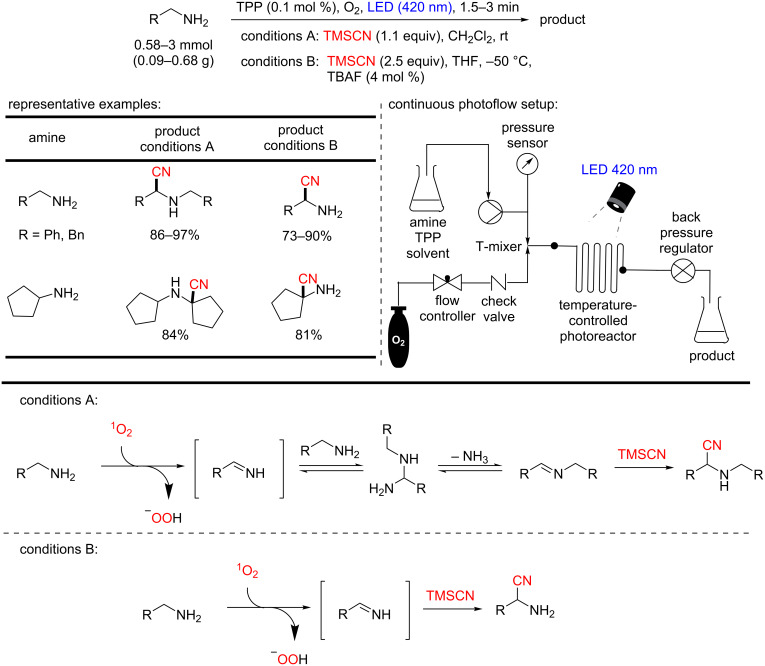
Photocyanation of primary amines.

**Scheme 57:**
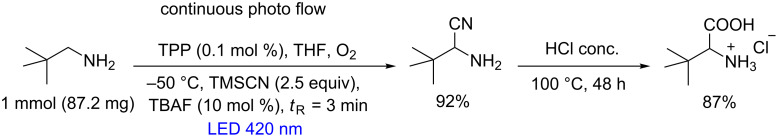
Synthesis of ᴅ,ʟ-*tert*-leucine hydrochloride.

In this regard, Seeberger and co-workers reported the primary/secondary amine oxidation under continuous-flow conditions using TPP as photocatalyst for singlet oxygen generation, and subsequently, the product imines were trapped with trimethylsilyl cyanide (TMSCN) for producing the α-aminonitriles (Scheme 56) [105]. A library of α-aminonitriles was produced by this methodology (conditions A). However, when primary amines were used, the authors observed an oxidative coupling between the amines and their respective *N*-substituted imines, which were trapped with TMSCN to afford the corresponding nitriles. The authors solved this problem by cooling the reaction to −50 °C and using 4 mol % of tetra-*n*-butylammonium fluoride (TBAF) as an activator of TMSCN (conditions B). Following this second protocol, the primary α-aminonitriles were rapidly prepared in relevant yields (up to 87%) and converted to the corresponding α-amino acids by hydrolysis of the nitrile (Scheme 57).

**Scheme 58:**
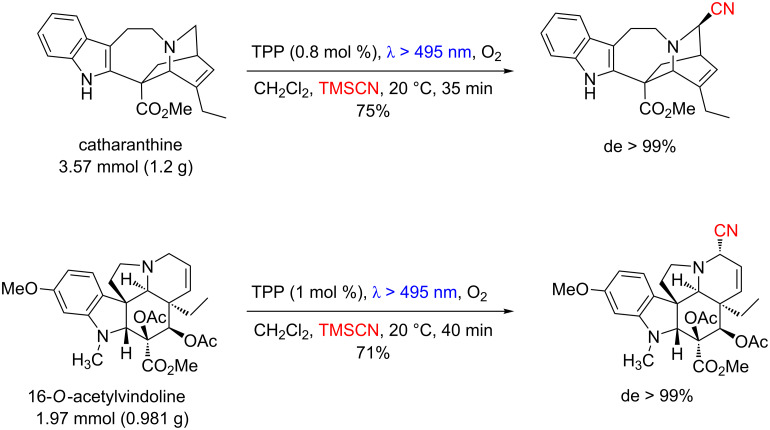
Photocyanation of catharanthine and 16-*O*-acetylvindoline using TPP.

Ferroud’s group showed that the α-photocyanation of amines can be efficiently applied in complex molecules. The authors reported a highly regio- and diastereoselective photocyanation of both catharanthine and 16-*O*-acetylvindoline alkaloids with TMSCN and using TPP as photocatalyst (Scheme 58) [106–107].

**Scheme 59:**
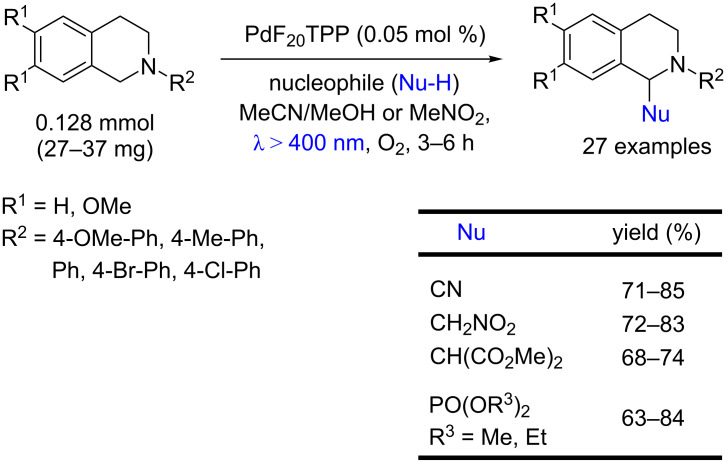
Photochemical α-functionalization of *N*-aryltetrahydroisoquinolines using Pd-TPFPP as photocatalyst.

Che and co-workers showed that a variety of nucleophiles can be used for the α-functionalization of *N*-aryltetrahydroisoquinolines using a low Pd-TPFPP loading. This photocatalyst provided the α-aminonitriles in 71–85%, β-nitroamines in 72–83%, β-diester amines in 68–74%, and α-amino phosphonates in 63–84% yields (Scheme 59) [97].

**Scheme 60:**
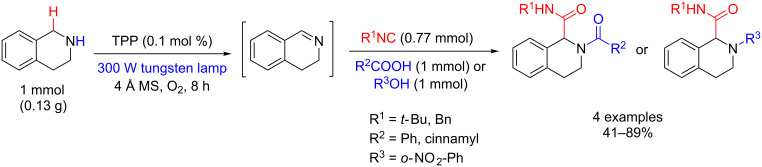
Ugi-type reaction with 1,2,3,4-tetrahydroisoquinoline using molecular oxygen and TPP.

**Scheme 61:**
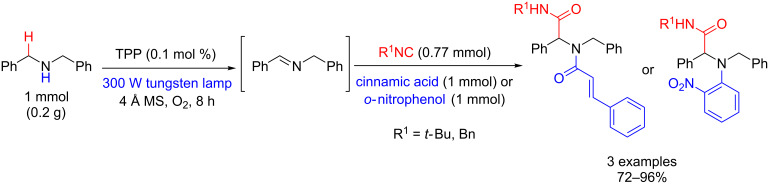
Ugi-type reaction with dibenzylamines using molecular oxygen and TPP.

The Ugi-type multicomponent reactions between imines, carboxylic acids, and isocyanides, and Mannich-type reactions between iminium and carbonyl groups have found many applications in organic synthesis [108]. Che’s group employed the methodology of oxidation of an amine with singlet oxygen to produce an imine, which was used as a substrate in the Ugi-type reaction. Thus, the oxidations of both 1,2,3,4-tetrahydroisoquinoline and dibenzylamine using TPP were carried out with high yield and selectivity, and the Ugi products were obtained after removal of the solvent and direct addition of isocyanide and carboxylic acid. The Ugi products were obtained in 41–89% and 72–96% yields from 1,2,3,4-tetrahydroisoquinoline (Scheme 60) and dibenzylamine (Scheme 61), respectively [102].

**Scheme 62:**
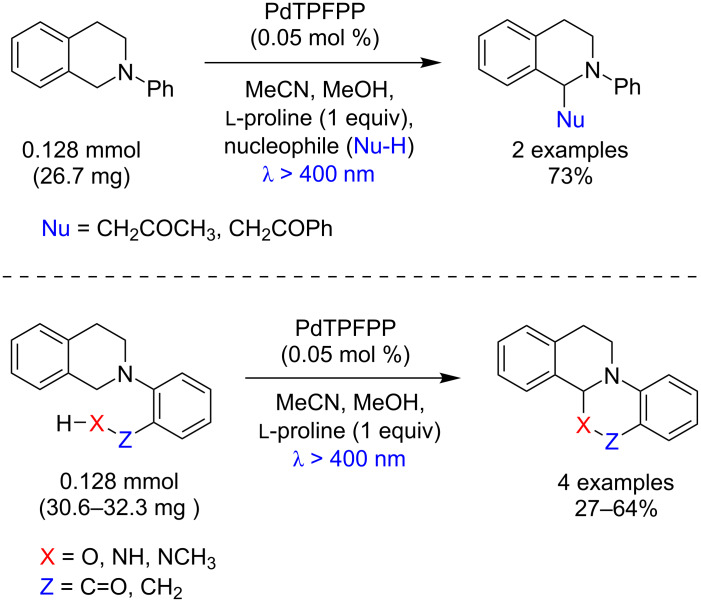
Mannich-type reaction of tertiary amines using PdTPFPP as photocatalyst.

Furthermore, Che and co-workers obtained a wide range of Mannich-type products by coupling *N*-aryltetrahydroisoquinoline, ketones, and ʟ-proline using a low PdTPFPP loading (Scheme 62) [97].

**Scheme 63:**
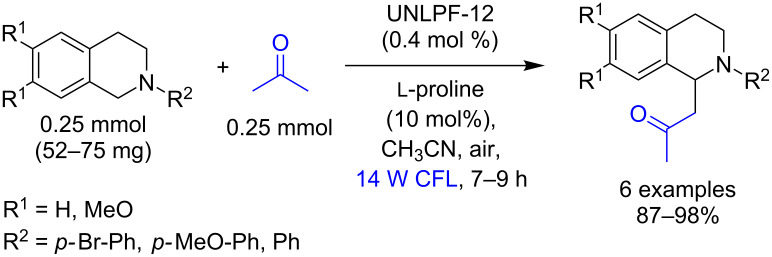
Oxidative Mannich reaction using UNLPF-12 as heterogeneous photocatalyst.

Zhang and co-workers demonstrated that the MOF-catalyst (UNLPF-12) can also be used for the Mannich reactions. The authors reported that the coupling between *N*-aryltetrahydroisoquinolines and acetone using visible light and UNLPF-12 afforded the Mannich products in 87–98% yields (Scheme 63) [40].

**Scheme 64:**
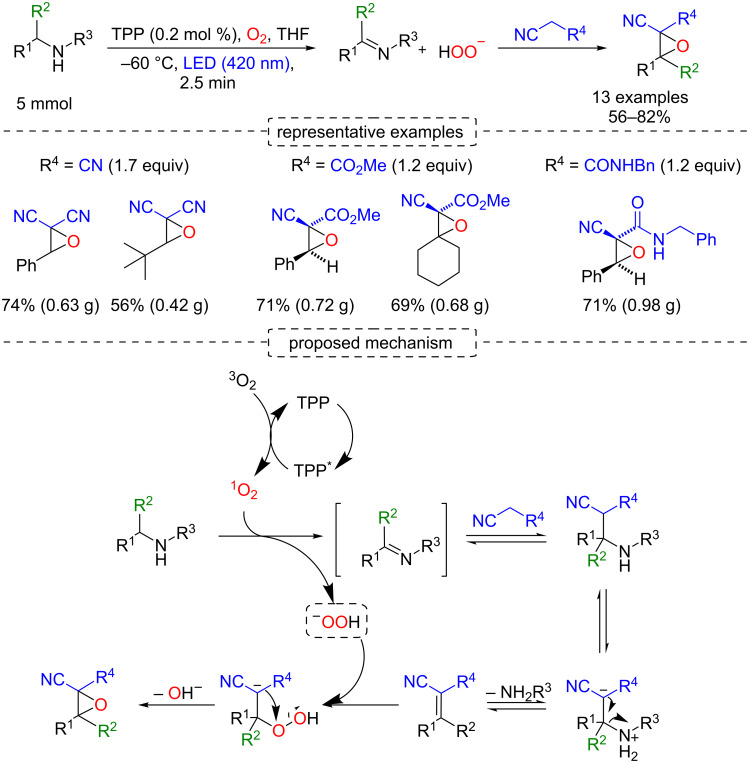
Transformation of amines to α-cyanoepoxides and the proposed mechanism.

As previously shown (Scheme 50), the oxidation of amines to imines by singlet oxygen furnishes hydrogen peroxide as a byproduct. In 2014, Seeberger and co-workers used this byproduct, in a continuous-flow approach, as an epoxidation agent of an electron-deficient olefin intermediate, which was formed by deaminative Mannich coupling between the imine and nucleophiles such as malononitrile and methyl cyanoacetate (Scheme 64) [109].

Overall, a variety of additional examples of porphyrin-photocatalyzed heteroatom oxidations are continuously under development, and we have highlighted herein the most relevant in the authors’ opinion.

## Conclusion

As demonstrated in this review, porphyrin derivatives have gained attention in preparative organic synthesis in the last 10 years with growing applications in photocatalysis. Relevant chemical transformations have been reported with scalability, which makes porphyrin chemistry more valuable and with potential for further preparative and industrial applications. Many challenges must still be solved in terms of the availability of these photocatalysts to make them cost-competitive; however, the very low loading of these compounds (less than 0.5–1 mol %), high TON and easy recovery can be considered important advantages. For the authors of this review, porphyrin photochemically mediated transformations in organic synthesis are definitively a very important field for further exploration in both single electron and energy transfer.
